# Innovative Self‐Powered Sensing: Potential of Fabrigami and Electrospun Nanofiber‐Based Triboelectric Nanogenerator for Joint Biomechanics Monitoring

**DOI:** 10.1002/smll.202506363

**Published:** 2025-09-27

**Authors:** K. R. Sanjaya D. Gunawardhana, Zhou Fang, Garrett B. McGuinness, Luz Alejandra Magre Colorado, Sonal Santosh Baberwal, Waseem Ahmad Wani, Brian J. Rodriguez, Robert O'Connor, Ciara Smullen, Tomás E. Ward, Shirley M. Coyle

**Affiliations:** ^1^ School of Electronic Engineering Dublin City University Glasnevin, Dublin 9 Ireland; ^2^ Insight Research Ireland Centre for Data Analytics Dublin City University Glasnevin, Dublin 9 Ireland; ^3^ School of Mechanical Engineering Dublin City University Glasnevin, Dublin 9 Ireland; ^4^ School of Physics and Conway Institute University College Dublin Belfield, Dublin 4 Ireland; ^5^ School of Physical Sciences Dublin City University Glasnevin, Dublin 9 Ireland; ^6^ School of Computing Dublin City University Glasnevin, Dublin 9 Ireland

**Keywords:** electrospinning, fabrigami, origami engineering, rehabilitation, self‐powered sensing, textile engineering, wearable electronics

## Abstract

This work presents a high‐performance, self‐powered triboelectric nanogenerator (TENG) embedded into an origami‐inspired fabric (“fabrigami”) structure for real‐time joint biomechanics monitoring. The device consists of electrospun polyvinylidene fluoride (PVDF) as the electronegative layer and silver‐doped cellulose acetate (Ag‐CA) as the electropositive layer, with enhanced surface charge density and mechanical durability. The fabrigami architecture amplifies contactseparation dynamics, enabling efficient detection of movements during joint motion while preserving conformability and air permeability. The optimized TENG based on 1.5% Ag in CA against PVDF exhibits remarkable electrical output characteristics, including an open‐circuit voltage of 155.9 V, short‐circuit current density of 8.134 mA m^−2^, and transferred charge density of 65.62 µC m‐, with an instantaneous peak power density of 0.029 W m^−2^ achieved through an 11 MΩ external load resistance. The power conversion efficiency is 4.6–92.8% for 100–5 µm elastic compression of electrospun samples under 10 N, 2 Hz actuation. Sensor stability is observed over 15 000 cycles. The fabrigami knee sleeve includes a Bluetooth‐enabled microcontroller transmitting real‐time motion data wirelessly to measure joint angles and distinguish movement activities. This work demonstrates a novel strategy combining material innovation (Ag‐CA nanofibers) with structural configurability to create a breathable and power‐autonomous smart textile.

## Graphical abstract

Novel “fabrigami” sensor is developed to monitor joint movements in real time. This device combines an origami‐inspired fabric with self‐powered textile sensors to convert movement into electrical signals. The sensors are based on self‐powered triboelectric nanofibers and connected to a wireless microcontroller to transmit human movement data to mobile devices. The device is capable of distinguishing between walking, running, and other activities, showing promise for home‐based rehabilitation and injury prevention in the future.

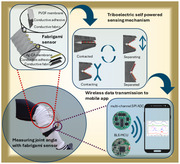

## 1 Introduction

Quantitative analysis of human kinematics through biomechanical measurements serves as a crucial tool for human health and performance evaluation. The assessment of joint biomechanics (JBM) enables healthcare providers to create personalized treatment and rehabilitation programs for patients whose movement patterns are impacted by osteoarthritis or injury recovery or post‐surgical conditions of the knee or hip. Beyond clinical applications, assessing biomechanics is fundamental to sports science, as well as comprehending and evaluating body mechanics in order to refine techniques, thereby enhancing athletic performance while at the same time reducing injury risks.

The accuracy of biomechanical assessments has improved significantly through technological progress, which replaced basic goniometers with advanced motion capture systems. Modern motion‐capturing systems deliver detailed data on the angles, speed, and frequency of human movements, which are essential for therapeutic and performance‐enhancement applications. However, they come with significant drawbacks, including the need for a substantial financial investment in their purchase, operation, and maintenance. Additionally, these systems require considerable processing power and must be set up in a controlled environment. Furthermore, limiting their portability and versatility for use in different settings makes them challenging to use for the improvement of users’ day‐to‐day activities.^[^ ^1^ ^]^

Inertial measurement units (IMUs) are predominantly used for motion tracking in recently developed wearable sensing applications due to their ability to capture dynamic movements and orientations accurately. IMUs typically consist of accelerometers, gyroscopes, and sometimes, magnetometers. Moreover, these sensors provide critical information, such as linear velocity, angular acceleration, and orientation, which are essential for detecting movement patterns, posture changes, and shifts in body orientation. Sensor fusion techniques, such as the Kalman filter, are often employed to enhance the accuracy of IMUs.^[^ ^2^ ^]^ These methods significantly reduce noise in the sensor signals and improve the reliability of the data collected by combining the information from multiple sensors. Recently, long short‐term memory neural networks, along with IMU sensors, have provided a high degree of precision for long‐term applications.^[^ ^3^ ^]^ However, despite these advances, the long‐term accuracy of IMUs is still limited due to sensor drift, which accumulates over time, leading to gradual errors in the measurement of orientation and position.^[^ ^4^, ^5^ ^]^ IMUs are not specifically designed to measure force or pressure‐related data, such as joint loads or the impact forces during walking or running.

Recent developments in sensor technology have introduced triboelectric nanogenerators (TENG) as a promising alternative for assessing human joint movement.^[^ ^6^, ^7^, ^8^, ^9^, ^10^ ^]^ TENGs use the triboelectric effect to generate electrical signals from mechanical motion, offering unique advantages such as high sensitivity, lightweight, flexibility, and easy fabrication over traditional systems. Unlike advanced motion capture systems, TENGs provide a more accessible and cost‐effective solution for wearable applications in both clinical and athletic environments.^[^ ^11^ ^]^

TENGs operate through contact electrification and electrostatic induction, where charges are transferred via an external electrode and subsequently through a load, generating a detectable signal. The sensitivity and accuracy of TENG‐based sensors are significantly influenced by the triboelectric substrate. Advancements in substrate design are crucial for optimizing sensor performance, including improvements in surface morphology and material composition to enhance the triboelectric effect.^[^ ^12^ ^]^ Additionally, optimizing the efficiency of charge transfer between the triboelectric layers is essential for maximizing electrical output,^[^ ^13^, ^14^ ^]^ requiring careful consideration of interface properties and material interactions. Furthermore, ensuring effective mechanical movement between the triboelectric layers is crucial, as it directly impacts the energy conversion efficiency.^[^ ^15^ ^]^ Collectively, these factors are vital in advancing the performance of TENG‐based sensors.

Since Wang's research group first introduced the concept in 2012, significant advancements have been made in TENG technology, particularly in wearable applications for detecting basic human activities, such as gait analysis,^[^ ^16^ ^]^ hand and finger movement detection,^[^ ^17^, ^18^ ^]^ body gesture recognition,^[^ ^19^ ^]^ physical exercise monitoring,^[^ ^20^, ^21^ ^]^ heart rate monitoring,^[^ ^22^ ^]^ and respiratory monitoring.^[^ ^23^, ^24^ ^]^ In recent years, experimental studies have explored the use of TENGs for upper and lower limb rehabilitation,^[^ ^25^ ^]^ muscle function recovery,^[^ ^26^ ^]^ pulmonary rehabilitation,^[^ ^27^ ^]^ and other applications, including neck injury rehabilitation caused by poor posture, accidents, or prolonged computer use.^[^ ^7^, ^9^ ^]^ Pandey et al. highlight that despite their precision, efficiency, and sensitivity for wearable JBM and rehabilitation applications, TENGs still face significant challenges.^[^ ^9^ ^]^ Many TENG‐based sensors used for rehabilitation are currently bulky and heavy, which hinders their practicality for real‐world use.^[^ ^25^, ^28^ ^]^ Moreover, the accurate detection of low‐frequency, low‐force movements, crucial for capturing detailed parameters like speed, frequency, and joint angles, remains an underexplored area in the context of TENG sensors. This work demonstrates the potential of electrospun‐based TENGs that are suitable for textile integration within a garment to measure such parameters related to joint biomechanics.

As previously discussed in our work, the use of electrospun membranes has been instrumental in enhancing the charge density, reducing the thickness and increasing charge transfer performance of TENG devices.^[^ ^11^ ^]^ These membranes offer a high surface area and customizable porosity, which improve charge generation and sensitivity, crucial for energy harvesting and sensing applications in wearable and biomedical devices.^[^ ^29^, ^30^, ^31^ ^]^ Among electrospun TENG materials, Polyvinylidene difluoride (PVDF) has been widely investigated as highly tribonegative and also has inherent piezoelectric properties.^[^ ^32^, ^33^ ^]^ Cellulose, being one of the most abundant polymers, has gained attention as both a functional material and filler in wearable electronics. Bai et al. utilized electrospun cellulose acetate (CA) modified with carbon nanotubes and combined with PVDF membranes in a contact‐separation configuration, achieving a sensitivity of 3.03 V kPa^−1^ up to 6.8 kPa and 0.11 V kPa^−1^ up to 65 kPa, demonstrating that CA's performance can be enhanced by incorporating conductive filler materials.^[^ ^34^ ^]^ Building on this foundation, in our research, we successfully incorporated silver (Ag) nanoparticles into the CA precursor, resulting in a significantly higher sensitivity of 11.667 ± 0.505 kPa^−1^ in the 0–6.25 kPa range and 5.157 ± 0.128 kPa^−1^ in the 6.25–18.75 kPa range. Furthermore, we have demonstrated the feasibility of using the developed TENG device for energy harvesting applications.

Origami, an ancient Japanese art form centered on folding paper to craft different shapes, has long been esteemed for its blend of simplicity and intricate design. Initially, it was used for art and entertainment, and recently, it has been gaining attention as a contemporary method for investigating geometry and engineering principles.^[^ ^35^ ^]^ Inspiring on this technique, fabrigami has emerged as an inventive adaptation, utilizing the same folding methods with pliable, resilient fabrics.^[^ ^36^ ^]^ Furthermore, origami structures can be permanently thermally imprinted into synthetic fabrics such as polyester, nylon, and similar polymer‐based materials. This heat‐setting technique should be carefully selected based on the thermal properties of these synthetic materials. Primarily, the process should promote molecular reorganization by applying temperatures above the material's glass transition temperature but below its melting point. This advancement facilitates the creation of adaptable, long‐lasting structures with potential applications in fields like soft robotics,^[^ ^37^, ^38^, ^39^ ^]^ wearable technology,^[^ ^40^, ^41^ ^]^ and adaptive materials.^[^ ^42^, ^43^ ^]^

Extending this exceptional sensitivity with Ag‐modified CA (Ag‐CA), we developed, for the first time, a fully fabric‐based sensor for measuring JBM, utilizing advanced fabrigami technology. This novel sensor addresses key limitations found in previous designs by offering a lightweight, highly flexible, and wearable solution. Furthermore, we have successfully demonstrated the sensor's ability to capture comprehensive JBM data, such as knee angle (to the nearest 10 degrees), distinguishing different movement patterns such as walking, running, stair climbing, stair descending and squatting exercises. These advancements mark a significant step toward overcoming existing challenges in TENG‐based sensors, offering enhanced performance for real‐time, wearable JBM in the future.

## 2 Results and Discussion

**Figure 1:**
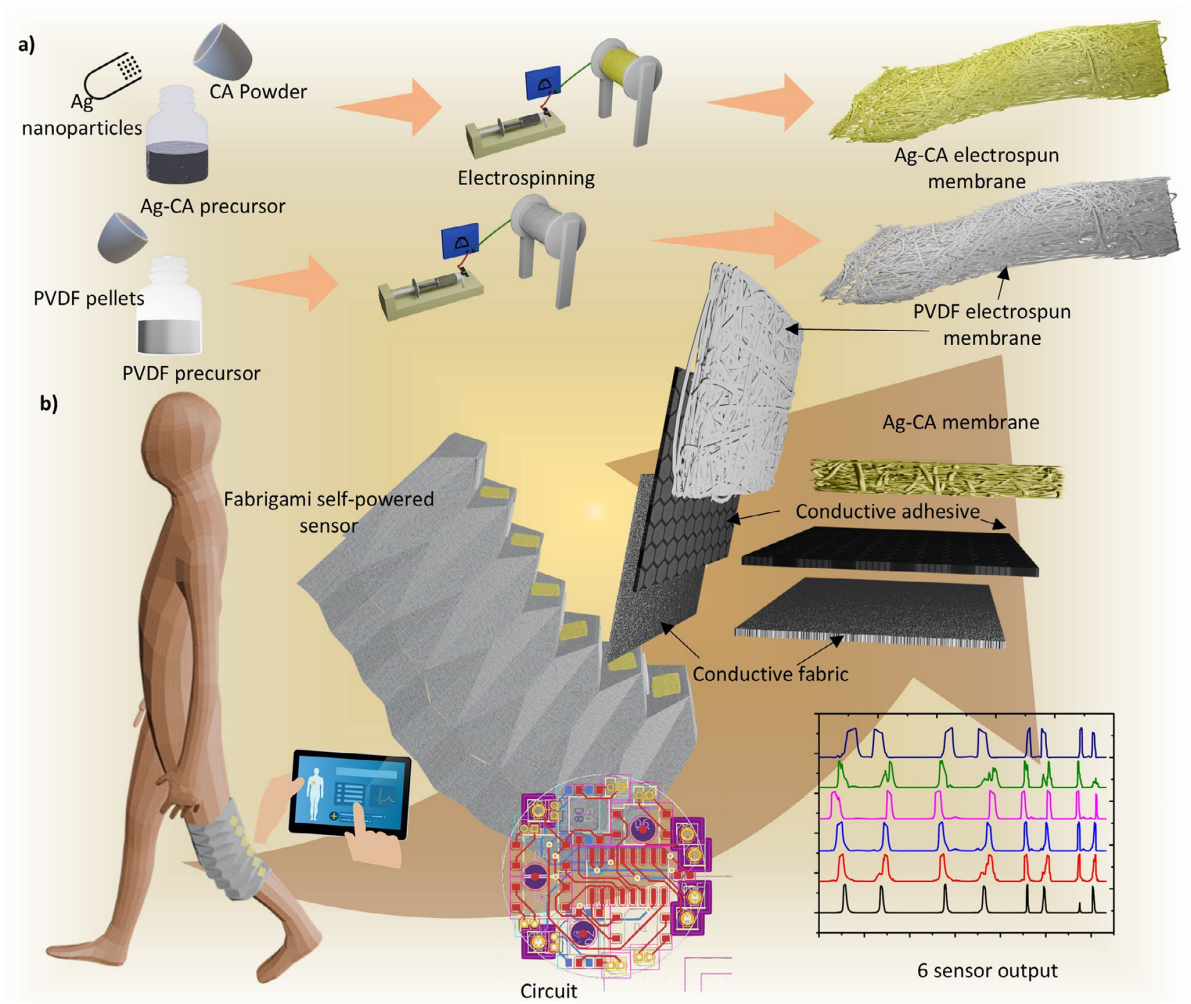
Fabrigami JBM monitoring sensor architecture: a) Fabrication process of electrospun membranes, and b) Application and data collection mechanism.

In **Figure** **1**, the fabrication process and the proposed concept of our final device are illustrated. The figure comprises two main components: i) the fabrication process and ii) the application and data collection mechanism. The electrospun membranes of Ag‐CA and PVDF have been employed as the tribopositive and tribonegative layers, respectively. Six sensors made with Ag‐CA and PVDF and attached Shieldex Zell RS piece as electrode were incorporated into the opposite sides of the valleys in the developed origami structure, as detailed in Section 3.4. The electrospun membranes were affixed to the electrodes using 3 M 9713XYZ tape. These 6 sensors are positioned behind the knee and, knee flexion causes the mountains of the fabrigami structure to close sequentially from top to bottom. This progressive contact‐separation mechanism generates electrical signal output in a pattern that correlates with the specific angle of knee flexion, enabling precise angle detection through the sensor activation sequence.

The results of this fabrication process demonstrate the successful integration of the materials into the fabrigami structure, leading to a functional device capable of efficiently monitoring JBM. The use of Ag‐CA and PVDF electrospun membranes provided enhanced triboelectric performance, evident from the electrical characterization and self‐powered sensing data (Sections 3.2 and 3.3) collected during the application. The findings also indicate that the choice of materials and the structural design play a crucial role in optimizing the device's performance, aligning with similar trends observed in previous studies (see Note S1, Supporting Information for comparative analysis of results from previous literature). These results underscore the potential of our approach in advancing the development of flexible and efficient triboelectric devices. Further discussion on the implications of these findings and their comparison with existing literature is provided in subsequent sections.

### 2.1 Material Characterization

**Figure 2:**
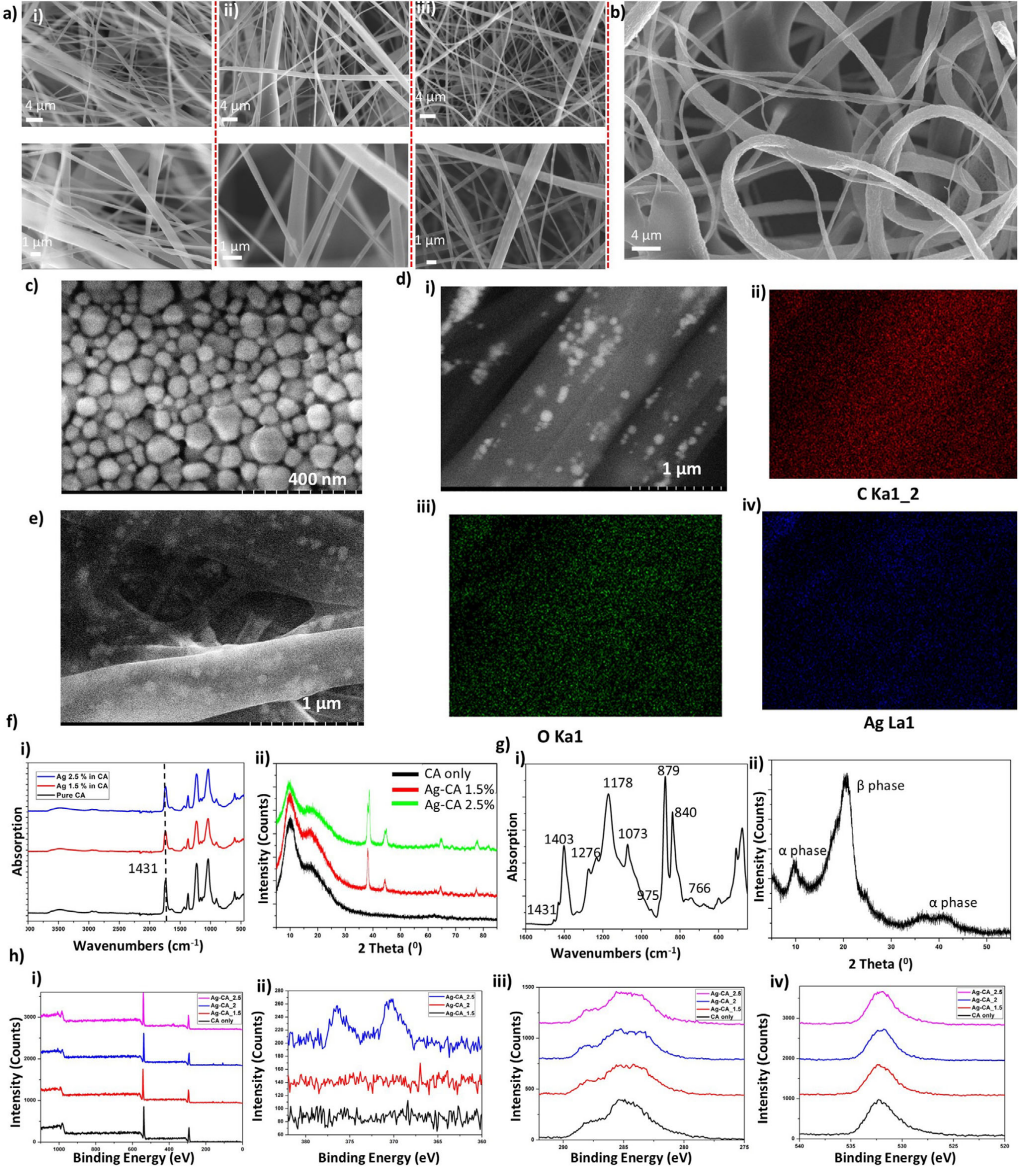
Material characterization of Ag‐CA and PVDF samples: a) SEM images of electrospun i) pristine CA, ii) Ag 1.5% in CA, and iii) Ag 2.5% in CA. b) SEM image of electrospun PVDF sample, c) high magnification image of Ag nanoparticles, d) EDS mapping with 1.5% Ag in CA i) high magnification image, EDS color maps of ii) carbon, iii) oxygen and iv) silver, e) high magnification image of Ag 2% in CA f‐i) FTIR and ii) XRD analysis of CA and Ag‐CA g‐i) FTIR and ii) XRD analysis of PVDF, h) XPS results of Ag‐CA samples i) survey scan, ii) silver 3d scan, iii) carbon 1s scan and iv) oxygen 1s scan.

The morphology of the developed layers was initially examined using scanning electron microscopy (SEM). **Figure** **2**a‐i–iii presents SEM images of pure CA, 1.5% Ag‐CA, and 2.5% Ag‐CA, respectively. Initially, we achieved bead‐free electrospun fibers by optimizing the electrospinning parameters. Detailed fiber diameter distribution graphs are provided in Note S2 (Supporting Information). Incorporating 1.5% Ag into pure CA did not significantly alter the average fiber diameter (pure CA: 515 ± 9.39 nm; 1.5% Ag‐CA: 516 ± 5.41 nm). However, increasing the Ag concentration to 2.5% significantly reduced the average diameter to 415 ± 6.61 nm. The reduction in fiber diameter is attributed to the increased conductivity of the electrospun precursor, which enhances the electrostatic repulsion and Coulombic forces at the needle tip, leading to a more pronounced elongation of the jet and a more stable formation of the Taylor cone. Furthermore, higher Ag content has improved the rough fibers compared with pristine CA.

PVDF is one of the most commonly used tribonegative materials with unique piezoelectric properties. The SEM image in Figure 2b depicts the morphology of the electrospun PVDF sample, revealing the formation of a well‐defined fibrous mat with a porous structure. The fibers exhibit an average diameter of 1.741 ± 0.103 µm, with the overall structure appearing stable and with minimal bead formation. Figure S3 (Supporting Information) presents the energy‐dispersive X‐ray spectroscopy (EDS) elemental maps for PVDF, where the color‐coded distribution confirms the presence of fluorine (F) uniformly throughout the fibers.

Having established the fiber morphology, we next examined nanoparticle distribution and chemical states to confirm successful incorporation of Ag into the CA matrix. The presence of Ag nanoparticles is confirmed by EDS analysis in Note S2 (Supporting Information), Figure S2 (Supporting Information) verifying the successful incorporation of Ag within the fibers. Commercial Ag nanoparticles were imaged separately, revealing an average diameter of 53.98 ± 2.47 nm and were randomly distributed within the CA matrix (Figure 2c,d) (Note S2, Supporting Information). A high‐magnification image illustrating the dispersion of 1.5% Ag nanoparticles within the matrix is provided in Figure 2d‐i. Furthermore, elemental maps analysis confirms the presence of Ag in the CA matrix, along with characteristic signals from carbon (C) and oxygen (O), consistent with the composition of cellulose acetate (Figure 2d‐ii–iv). According to Figure 2e when the Ag doping increases from 1.5% to 2% we observed an increase in the Ag nanoparticles distribution throughout the fibers.

Based on the FTIR analysis shown in Figure 2f‐i, incorporating Ag into CA did not result in observable differences in the FTIR spectra between pure CA and Ag‐doped CA samples. The absence of new or shifted FTIR peaks indicates that the Ag particles are likely not chemically bonded to the CA functional groups. Instead, the Ag may be physically dispersed within the polymer matrix without forming solid interactions with the CA's molecular structure, which would have been detectable by FTIR. In contrast, the X‐ray diffraction (XRD) analysis shown in Figure 2f‐ii reveals a clear difference in patterns between pure CA and Ag‐CA. The XRD patterns of pristine CA show the characteristic two humps centered at ≈ 2θ = 12° and 18°, indicative of its predominantly amorphous structure.^[^ ^44^ ^]^ Upon silver incorporation, this amorphous profile remains, but additional sharp diffraction peaks appear at 2*θ* ≈ 38.2°, 44.5°, 64.8°, and 77.4°, which correspond to the (111), (200), (220), and (311) planes of face‐centered cubic (fcc) metallic Ag (JCPDS No. 04‐0783).^[^ ^45^ ^]^ The intensity of these Ag peaks increases systematically with Ag loading (1.5% → 2.5% wt), consistent with higher nanoparticle content. While CA's amorphous phase is not converted to a crystalline phase, the incorporation of crystalline Ag NPs modifies the composite's overall diffraction profile, reflecting the dual‐phase (amorphous CA + crystalline Ag) nature of the material.

PVDF materials are known to exhibit various crystalline phases, with the polarization properties primarily attributed to the high content of the β phase. Previous studies, such as the work by Singh et al., have demonstrated that optimizing electrospinning parameters can significantly enhance the β phase content in PVDF nanofibers.^[^ ^32^ ^]^ Building on these findings, we selected and adjusted the electrospinning parameters to align with the constraints of our setup. As shown in Figure 2g‐i, the formation of the β phase is evident, which we quantified using the Lambert‐Beer law. By calculating the absorbance at 766 cm^−1^ (A_α_) and 840 cm^−1^ (A_β_), the β phase fraction was determined to be 75.75% (details provided in Note S3, Supporting Information). XRD analysis (Figure 2gii) further corroborates the significant presence of the β phase.^[^ ^33^ ^]^ While literature suggests that specific stretching effects,^[^ ^46^ ^]^ corona poling^[^ ^47^ ^]^ or incorporating nanoparticles like LiCl, CNT, BaTiO_3_, and graphene,^[^ ^48^, ^49^ ^]^ can further enhance β‐phase content, reported values typically achieved 85–95% at most and complete(100%) conversion is rarely observed. In this work we opted to use pristine electrospun PVDF as a baseline for characterizing the Ag‐CA mat.

While FTIR results indicate that Ag does not chemically modify CA, the XRD data imply that Ag influences the physical structure and crystallinity of the material, potentially enhancing properties such as conductivity or mechanical strength in the Ag‐doped samples. To investigate the surface chemistry of Ag and its bonding state with CA, X‐ray photoelectron spectroscopy (XPS) analysis was conducted and provided in Figure 2h‐i–iv. Ag signal was below detection limit for a samples with less than 2.5% Ag loading. For the 2.5% Ag‐loaded sample, although the signal‐to‐noise ratio was low reflecting the proximity of Ag content to the XPS detection limit the Ag 3d_5/2_ and Ag 3d_3/2_ peaks exhibited two components. These indicate that the silver at the surface is predominantly in an oxidized state (binding energy ≈368 eV), with some metallic contribution (≈366 eV). The C 1s spectra were broad in all cases, revealing components corresponding to C─C/C─H bonds at 284.5 eV, C─O bonds at 286 eV, and a shoulder at ≈289 eV attributed to C═O functionalities.

**Figure 3:**
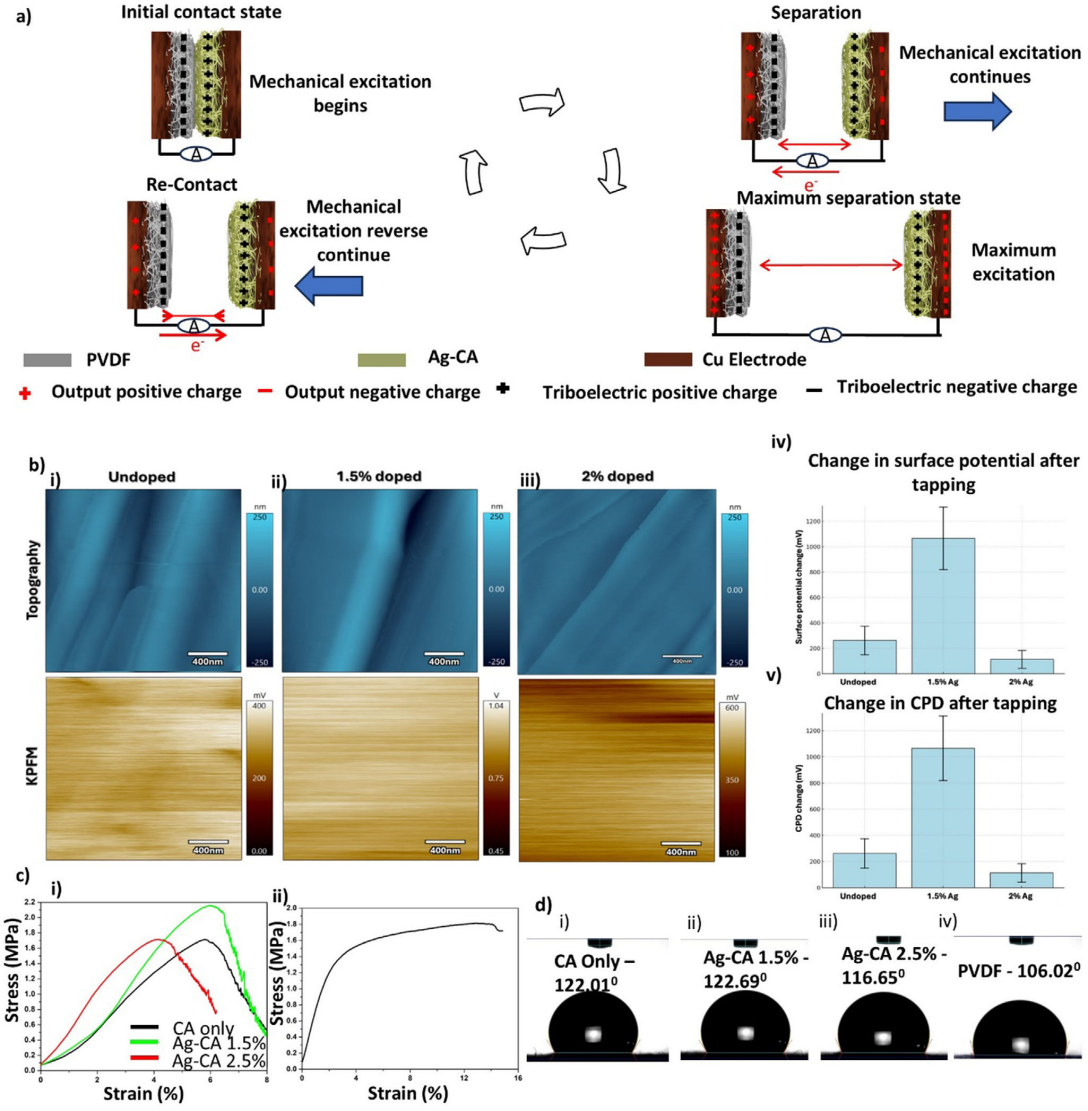
Working mechanism, KPFM analysis and mechanical characterization of samples. a) Working mechanism of Ag‐CA and PVDF sample based TENG b) Topography and KPFM of i) undoped, ii) 1.5%‐doped, and ii) 2%‐doped samples, iii) surface potential change after tapping, iv) CPD change after tapping c) Tensile property analysis of CA and Ag‐CA (i), and PVDF (ii). d) Contact angle measurements of CA (i), Ag 1.5% in CA (ii), Ag 2.5% in CA (iii), and PVDF (iv) electrospun samples

TENG devices operate on the principles of triboelectrification, which arises from the relative motion between two triboelectric materials and electrostatic induction between the conductive substrates attached to these materials. Although the exact mechanism remains unclear, it likely involves the transfer of charges, electrons, ions, or a combination of these factors.^[^ ^6^, ^11^, ^50^ ^]^ The triboelectric series categorizes materials based on their tendency to gain positive or negative charge upon contact.^[^ ^51^ ^]^ CA is moderately positive in this context, while PVDF is highly negative. The contact and separation between CA and PVDF result in CA acquiring a positive charge and PVDF a negative charge, driving electron flow between the attached electrodes and generating an alternating current. In the triboelectric series, Ag is positioned towards the negative side, and with the presence of Ag in the composite, the triboelectric contrast between Ag‐CA and PVDF will decrease because the composite becomes more negative due to the influence of Ag. The contact and separation mechanism is provided in **Figure** **3**a.

To further understand the surface charge behavior of the Ag‐CA samples Kelvin probe force microscopy (KPFM) measurements were carried out. Figure 3b shows the topography and the contact potential difference (CPD) value of the undoped, 1.5% Ag‐doped, and 2% Ag‐doped samples. Although the topography appears to be quite similar between samples, their CPD values showed significant differences. For the undoped sample, the CPD of the fiber was measured to be 423 ± 46 mV. In contrast, the 1.5% Ag‐doped sample exhibited a significantly higher CPD of 856 ± 102 mV.

To get further insights about the influence of Ag doping on the triboelectric effect, the samples were manually tapped with PVDF, and then KPFM measurements were retaken. The variations in CPD after tapping are presented in Figure 3b‐iv,v. Interestingly, after tapping, the 1.5% Ag‐doped sample showed the highest CPD, whereas the CPDs of the undoped and 2% Ag‐doped samples remained within the standard deviation after tapping. This trend aligns well with the results obtained from bulk triboelectric measurements. However, after tapping, the surface charge was observed to decay rapidly over time, with a decay of more than 70% in 24 min. This suggests that although doping enhances the initial surface potential, long‐term stability of the generated charge remains a challenge.

Based on KPFM results and high‐magnification SEM/EDS imaging, we interpret the behavior at and above 2.0 wt% Ag as approaching the percolation threshold of Ag within the CA matrix. Ag nanoparticle doping is expected to increase the effective permittivity of the composite due to enhanced interfacial polarization (Maxwell–Wagner–Sillars (MWS)) effects,^[^ ^52^ ^]^ which can improve surface charge storage under contact electrification. However, beyond a certain loading threshold, the formation of conductive pathways may result in increased leakage current and dielectric loss, as predicted by percolation theory which reduces effective dielectric response and triboelectric output. Similar behavior has been observed in silver–cellulose composites: for example, a cellulose nanofibril sponge with only ≈0.1 vol% Ag nanowires exhibits percolation‐driven electrical conductivity of ≈1.5 S cm^−1^,^[^ ^53^ ^]^ supporting our interpretation that the ≈2 wt% Ag content in our CA fibers (≈0.2–0.3 vol%) approaches a percolation threshold that reduces effective dielectric and triboelectric performance. Although percolation thresholds depend on filler shape and dispersion Ag nanowires can percolate at lower vol% than spherical nanoparticles This correlates with our observed decrease in open‐circuit voltage and transferred charge at higher Ag concentrations (2%>) (Section 3.2), suggesting that the percolation threshold may have been approached. Future work will include low‐frequency capacitance and dielectric loss measurements to further strengthen this analysis.

The mechanical properties of pristine CA and CA with 1.5% and 2.5% Ag are characterized in Figure 3c. An initial increase in maximum tensile stress is observed in 1.5% Ag‐CA than pristine, reaching 2.104 MPa at 6.3% strain,^[^ ^54^ ^]^ likely due to the formation of smoother, more directional fibers with the incorporation of Ag nanoparticles. However, as the Ag content increases, the maximum tensile stress decreases to 1.633 MPa at 4.2% strain. This reduction in mechanical strength can be attributed to the agglomeration of Ag nanoparticles at higher concentrations, which disrupts the uniformity of the fiber structure, leading to stress concentration points and a subsequent decrease in tensile strength. In contrast, the PVDF sample exhibits a maximum tensile strength of 1.815 MPa at approximately 13% strain. The contact angle measurements (Figure 3d) for pristine CA, 1.5%, and 2.5% Ag‐CA were determined to be 122.01°, 122.69°, and 116.65°, respectively. Pristine CA exhibits a high contact angle of 122.01°, reflecting its inherently hydrophobic surface. Upon the incorporation of 1.5% Ag nanoparticles, a slight increase in the contact angle to 122.69° is observed. However, further increasing the Ag concentration to 2.5% results in a decrease in the contact angle to 116.65°, indicating a reduction in hydrophobicity.

### 2.2 TENG Characterization

We utilized the distance‐dependent electric field (DDEF) model developed by Dharmasena et al.,^[^ ^55^, ^56^ ^]^ to theoretically simulate the charge transfer mechanism in our system, with results discussed in Note S4 (Supporting Information). The modified equations in Note S4 (Supporting Information) based on the parameters in our experiments, were used to evaluate and simulate the results for our selected materials.

**Figure 4:**
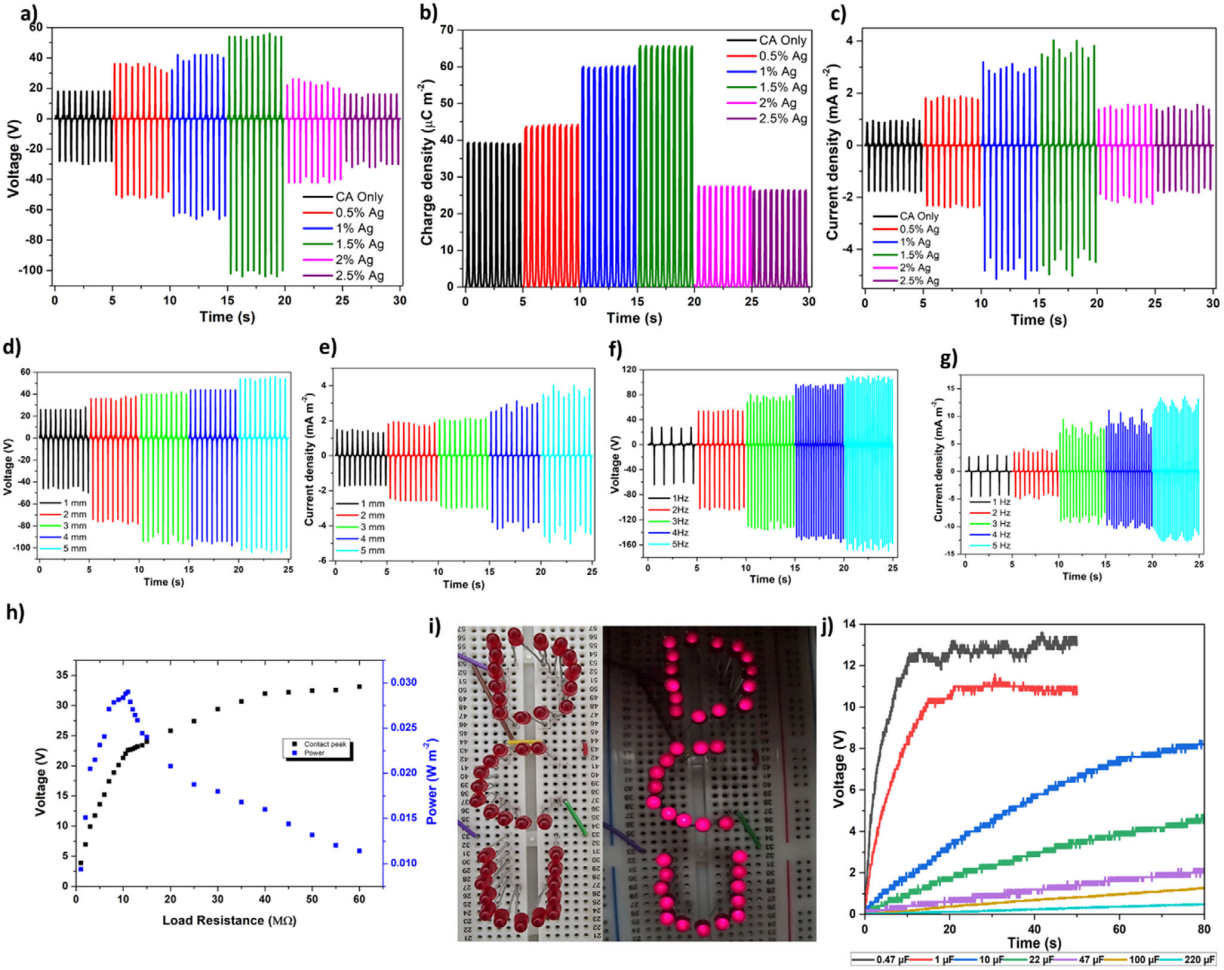
TENG performance evaluation: a) variation in voltage, b) charge, and c) current density concerning different Ag content in CA. Variation of d) voltage and e) current density output with respect to amplitude (frequency 2 Hz), variation of f) voltage and g) current density with respect to frequency (amplitude at 5 mm), h) power generation capability of the optimized TENG as a function of external impedance. i) Demonstration of LED lighting, and j) charging performance of various capacitors using hand tapping with the developed Ag‐CA and PVDF‐based TENG.

The experimental electrical characterization was conducted using the setup described in Note S5 (Supporting Information). A contact–separation mode TENG architecture was employed, applying a contact and separation force of 10 N, with a 5 mm amplitude and a frequency of 2 Hz. The initial electrical performance of the samples was characterized by measuring the open‐circuit voltage (*V*_OC_), transferred charge density (*Q*_SC_) and short‐circuit current density (*J*_SC_), as shown in Figure 4a,b,c, respectively. It was noted that the ascending (contact) cycle output is lower than the descending (separation) cycle output. According to Dharmasena, in contact and separation mode TENG devices, due to induced impulsive separation the separation peak can be greater than the contact peak.^[^ ^57^ ^]^ We observed variations between the separation and contact peak in some samples which may be attributed to the surface adhesion property variation of different electrospun samples. To reduce the effect of environmental conditions, all samples were characterized on the same day at normal room temperature and humidity. When paired with PVDF, the pristine CA exhibited a peak‐to‐peak *V*_OC_ of 46.4 V, a *J*_SC_ of 2.668 mA m^−2^, and a *Q*_SC_ of 39.051 µC m^−2^. Notably, the highest average peak‐to‐peak performance was observed with the 1.5% Ag‐CA sample in contact with PVDF, achieving a *V*_OC_ of 155.9 V, a *J*_SC_ of 8.134 mA m^−2^, and a *Q*_SC_ of 65.622 µC m^−2^ (**Figure** **4**a–c). This significant enhancement can be attributed to the optimized incorporation of Ag nanoparticles, which likely improved the surface charge transfer efficiency and enhanced the overall triboelectric performance. However, a further increase in Ag content to 2‐2.5% resulted in a reduction in output, which can be attributed to the agglomeration of Ag nanoparticles reaching the percolation threshold at higher concentrations. This agglomeration likely disrupted the distribution of surface charges and created charge leakages, leading to decreased efficiency in the triboelectric charge generation and transfer processes. Furthermore, an increase in conductivity might have disturbed the charge transfer mechanism, resulting in a lower outcome.^[^ ^58^ ^]^

Following the identification of the 1.5% Ag‐CA system as exhibiting the highest performance, all subsequent experiments were conducted using this optimized composition. Theoretical simulations revealed a positive correlation between output performance and both frequency and amplitude. To experimentally validate these findings within the limitations of our characterization setup, we systematically varied the amplitude and frequency. Increasing the amplitude from 1 to 5 mm at a constant frequency of 2 Hz led to a notable enhancement in device performance, with the *V*_OC_ rising from 72.2 to 155.9 V and the *J*_SC_ increasing from 3.093 to 8.134 mA m^−2^ (Figure 4d,e). Similarly, by increasing the frequency from 1 to 5 Hz at a fixed amplitude of 5 mm, the *V*_OC_ improved from 90 to 265.28 V, while the *J*_SC_ increased from 7.224 to 23.74 mA m^−2^ (Figure 4f,g). These results support the feasibility of detecting variable frequencies and amplitudes related to human joint movements.

To accurately determine the internal impedance, a range of load resistors were connected in a closed‐loop circuit, with voltage measured across the resistor terminals. Considering the average of contact cycle voltage (V) maximum instantaneous power density was calculated using *V* ^2^/(R*A), where *R* is the load resistance and A is the surface area of TENG device. Instantaneous peak power of 0.029 W m^−2^ was observed through an 11 MΩ resistor (Figure 4h). Bai et al. previously demonstrated the incorporation of CNTs into CA and PVDF electrospun membranes for TENG applications, achieving a maximum power density of 0.74 W m^−2^ with an 80 MΩ external resistor.^[^ ^34^ ^]^ Although our power density is lower, the reduced internal impedance achieved in our study enhances compatibility with modern electronic circuits, representing a significant step toward practical application. Also, we use the root mean square voltage to calculate the maximum average power, which resulted in 1.866 mW m^−2^ through an 11 MΩ resistor (Note S6, Supporting Information). We report a parametric estimate of power conversion efficiency for the wearable TENG: under test conditions (10 N, 2 Hz) and assuming 5–100 µm compression of the electrospun layers, the efficiency was found to vary from ≈93% (5 µm) to ≈4.6% (100 µm). Reported efficiencies are parametric estimates and are sensitive to micro‐compression (Note S7, Supporting Information). Previously reported TENG energy‐conversion efficiencies span a broad range due to device architecture and the evaluation method. Some previous benchmark designs using freestanding‐grating or rolling modes reported higher instantaneous efficiencies (≈55–85%) under specific operating assumptions.^[^ ^59^, ^60^, ^61^ ^]^ Recent experiments have quantified efficiencies around ≈25% for optimized devices, e.g., kinetic energy calculation and current integration (KECCI)‐based analysis yielding 24.89%,^[^ ^62^ ^]^ and ≈42.5% for high‐performance rotating architecture.^[^ ^63^ ^]^ Taken together, these reports explain the spread in literature values and place our parametric estimates (≈4.6–92.8% depending on micro‐compression) within the established landscape. To further examine the energy‐harvesting capability of the developed TENG architecture, the characterized 1.5% sample paired with PVDF was used to directly power and illuminate 38 light‐emitting diodes (LEDs) through contact–separation cycles induced by manual tapping, without any external power source (Figure 4i). Additionally, a circuit incorporating a full‐bridge rectifier was designed to charge a series of capacitors with different capacitances. Using the developed TENG and hand tapping, capacitors with values of 0.47, 1, 10, 22, 47, 100, and 220 µF were charged to 12.6 V (in 10 s), 10.5 V (in 15 s), 8 V (in 69 s), 4.6 V (in 75 s), 2.2 V (in 78 s), 1.3 V (in 78 s), and 0.5 V (in 80 s), respectively (Figure 4j).

### 2.3 Self‐Powered Sensing Evaluation

**Figure 5:**
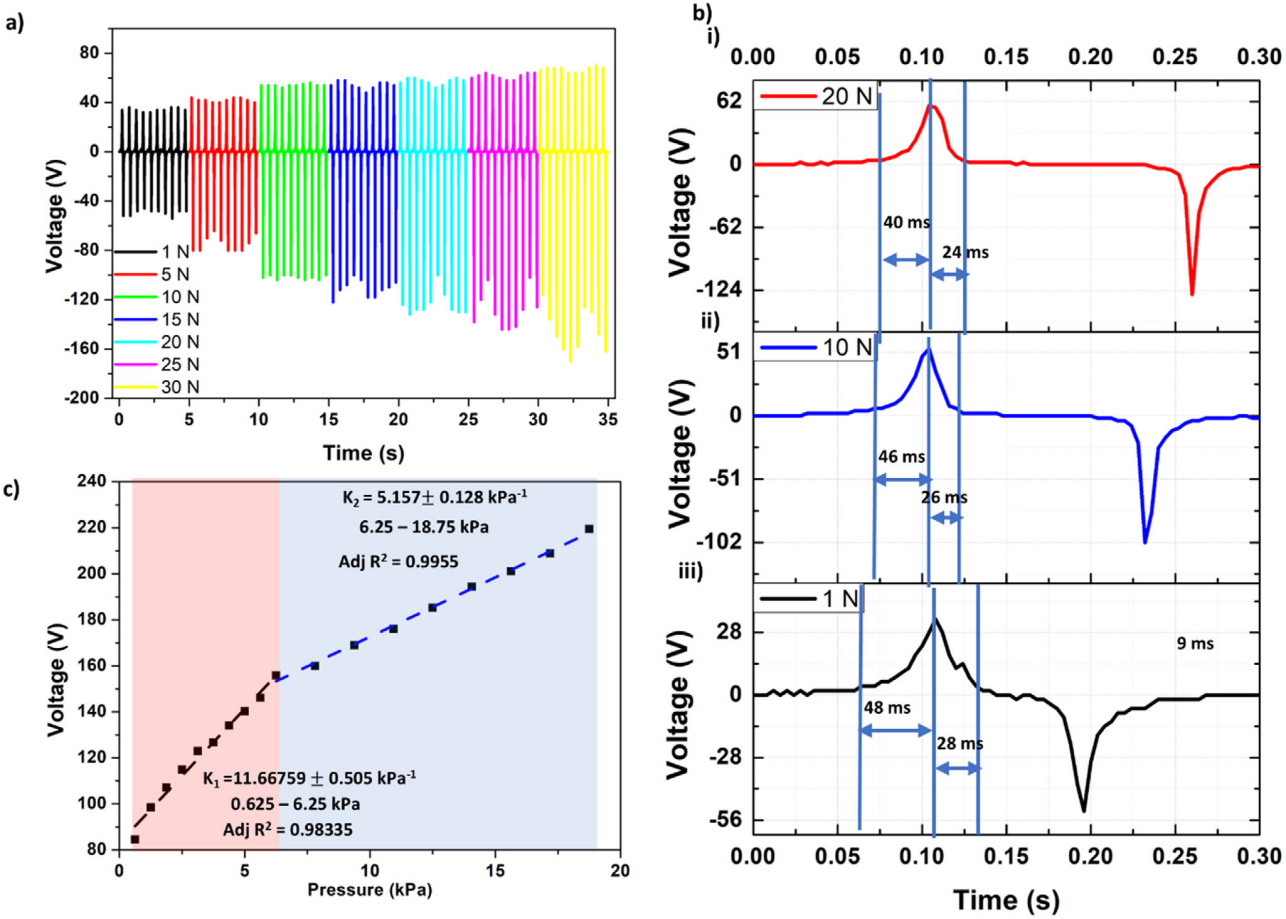
Self‐powered sensing performance evaluation: a) Variation in peak‐to‐peak voltage with applied force. b) Evaluation of response time and recovery time for forces of i) 20 N, ii) 10 N, and iii) 1 N. c) Linearity of voltage with applied pressure.

In this study, we aimed to explore the potential of an electrospun TENG substrate for detecting low‐pressure contacts with high sensitivity and accuracy, which can be elicited through the fabrigami structure. To investigate this phenomenon, we utilized a composite substrate comprising 1.5% Ag‐CA and PVDF and conducted a series of experiments to evaluate its pressure sensitivity, as illustrated in **Figure** **5**. Upon applying contact and separation forces ranging from 1 N to 30 N, we observed a corresponding increase in peak‐to‐peak voltage from 84.51 to 219.5 V, respectively (Figure 5a). Additionally, the response times in contact at 1 N, 10 N and 20 N were recorded as 48, 46, and 40 ms, while the recovery time was 28, 26, and 24 ms (Figure 5b). Further characterization with average peak to peak voltage at different pressure revealed that the sensor exhibits a pressure sensitivity of 11.667 ± 0.505. kPa^−1^ in the 0–6.25 kPa range, which decreases to 5.157 ± 0.128 kPa^−1^ in the 6.25–18.75 kPa range (Figure 5c). The sensor demonstrated stable performance over 15000 contact‐separation cycles under an applied force of 10 N at a frequency of 2 Hz (Note S8, Supporting Information). The high sensitivity rapid response at lower pressure levels and stability for long‐term use highlight the suitability of the developed sensor for JBM measurements, making it a promising candidate for applications in rehabilitation and sports injury prevention.

### 2.4 Fabrigami Structure Development

**Figure 6:**
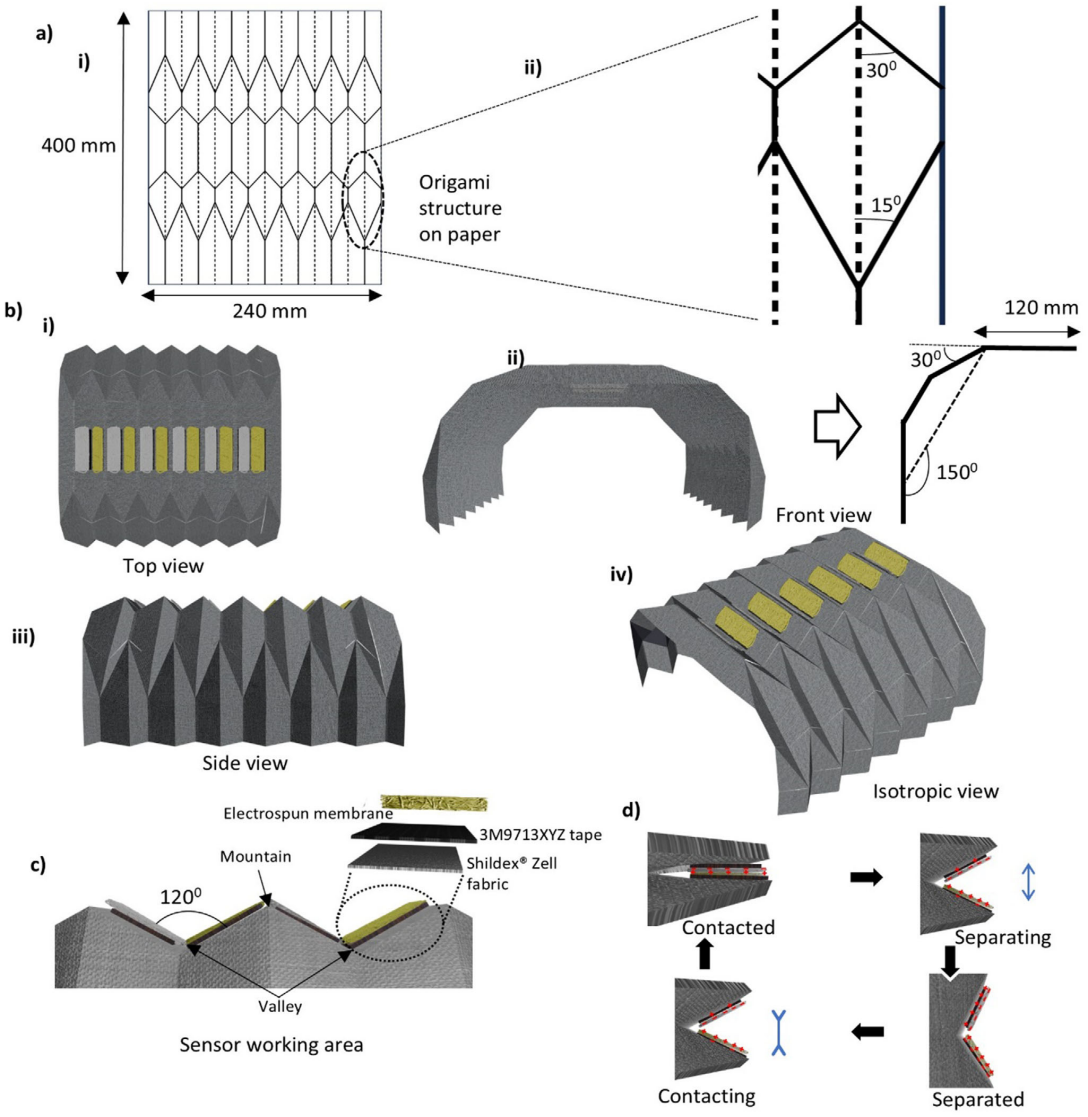
Fabrigami structure development: a‐i) Fabrication of the paper mold structure. ii) Explanation of different angles in the structure; b) Schematic view of the finalized Fabrigami structure, top view (i), front view (ii), side view (iii) and isotropic view (iv). c) Schematic of the sensor working area. d) Working principle of the newly developed sensor.

The development of the fabrigami structure commenced with the design of a 2D paper mold specifically tailored to capture the knee's JBM. The goal was to translate the intricate movements of the knee into a responsive fabrigami structure capable of enhancing the TENG's performance. We selected commercial white polyester fabric with 167.4 grams per square meter (gsm) to create this fabric‐based origami structure. This material was plain woven with a low thread count of 102, ensuring that the fabric possessed both the flexibility required for dynamic folding and the structural integrity needed to maintain the origami form under repeated stress.^[^ ^64^ ^]^ Polyester was chosen for its well‐documented mechanical properties, including high tensile strength, thermal stability, and resistance to environmental degradation. These properties make polyester an ideal candidate for applications where durability and resilience are paramount, particularly in wearable technologies. The 3D origami structure was formed by folding two pieces of paper into a complex configuration, as shown in **Figure** **6**a. The folding process utilized a pattern with solid lines on one side representing mountain folds and dotted lines on the other side indicating valley folds (Figure 6a‐vii). The mountain folds are the sharp ascending points on the structure while valley folds are the sharp descending points in the structure as shown in Figure 6a‐vii).

The choice of folding angles and patterns was not chosen arbitrarily; instead, it was based on a series of experimental trials using different paper molds. Selection of angles was conducted based on a model we prepared to understand the relationship between the knee diameter and the length and width of different parts. Note S9 (Supporting Information) summarizes all the equations that can be used to determine this length when the full width from mountain fold to adjacent valley fold is 15 mm. These trials aimed to identify the optimal angles and fold configurations to maximize the contact–separation cycles essential for the TENG's operation. The final design, depicted in Figure 6a‐i,ii, was selected for its ability to consistently produce distinct and efficient contact–separation events, a key factor in optimizing the triboelectric effect. We employed a thermal processing method to imprint the origami pattern onto the polyester fabric permanently. The fabric, sandwiched between the folded paper molds, was subjected to a controlled heating process at 140 °C for 20 min. This temperature was deliberately chosen to exceed the glass transition temperature (*T*g) of polyester, which is 68 °C, ensuring that the fabric could be softened sufficiently to conform to the mold's intricate folds. Upon cooling, the fabric retained the 3D origami structure, a result corroborated by previous studies showing that thermal imprinting at temperatures 130–150 °C can permanently alter the morphology of polymeric materials.^[^ ^65^, ^66^, ^67^ ^]^

Once the fabrigami structure was formed (Figure 6b‐i–iv), we selected mountain folds to attach six sensors on either side of each of these mountain folds(Figure 6c) only one side in first mountain fold and last mountain fold). An additional conductive layer was applied between the electrospun substrate and polyester fabric using Shieldex Zell RS fabric (0.02 Ω/□), known for its excellent conductivity and flexibility to enhance electrostatic induction. Ag‐CA layer was applied onto the conductive fabric with 3M9713XYZ tape to ensure robust and reliable electrical connections. On the opposite sides of each Ag‐CA layers, a sample of PVDF was attached using a similar technique. A conductive yarn (0.04 Ω cm^−1^) created connections from the Shieldex fabric and the conductive connectors to the circuit within the fabric substrate. At rest, the sensor's active area encompassed 300 mm^2^, providing a substantial surface for the contact–separation interactions critical to the TENG's operation. As shown in Figure 6d, the developed fabrigami structure facilitated consistent and effective contact‐separation cycles, crucial for the TENG's reliable performance. The sensors are numbered from bottom to top, with sensor 1 being the bottom‐most and sensor 6 being the top‐most.

**Figure 7:**
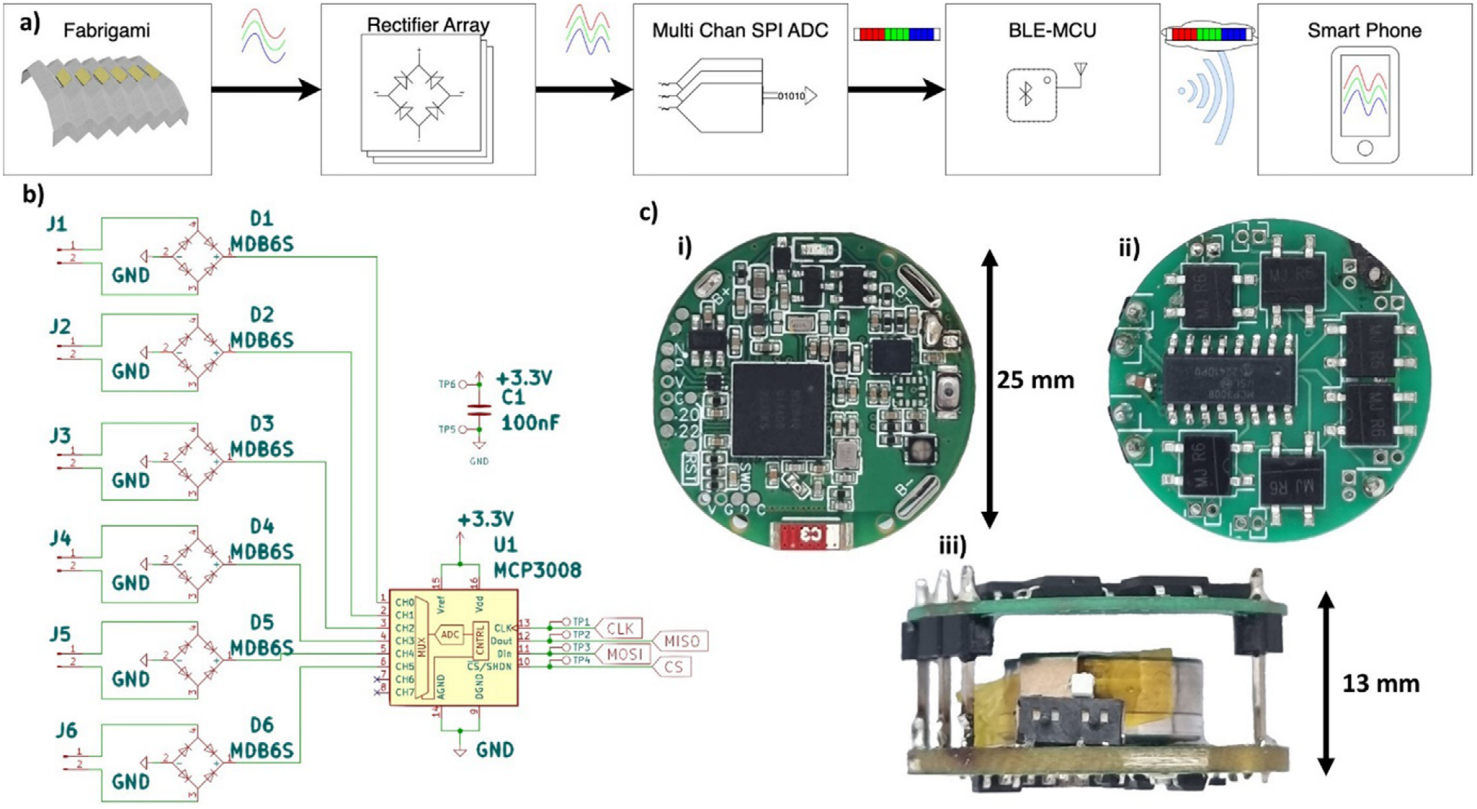
Flow chart of the data collection mechanism with the developed sensor a)including the fabrigami sensor, rectified array, multi‐channel SPI ADC, BLE‐MCU, and data collection with the smartphone, b) schematic diagram of rectified array and multi‐channel SPI ADC., c) images of final circuit i) top, ii) bottom and iii) side view.

The data acquisition process for the fabrigami sensor system is illustrated in **Figure** **7**a. Analog outputs from the six sensors are first processed through individual bridge rectifiers and then fed into an 8‐channel, 10‐bit analog‐to‐digital converter (ADC) with serial peripheral interface (SPI) (MCP3008), effectively mapping the signal within the 0–3.3 V input range. The digitized signals are transmitted via a Bluetooth enabled microcontroller unit (BLE‐MCU) (nRF52840), which integrates an onboard LSM6 6‐axis IMU and RGB LED, and communicates with a mobile app (Figure 7b). Complete sensor preparation process starting from folding paper to final sensor development is provided as Video S1 (Supporting Information).

The hardware is arranged in a compact, sandwich‐style configuration comprising two stacked PCBs with a VARTA 1454 rechargeable battery (0.3 Wh, 14 × 5.4 mm) embedded between them. Board 1 contains the BLE MCU and battery management circuitry, while Board 2 contains the ADC, six MDB6S bridge rectifiers, and lead pairs connecting to the fabric sensor. Communication between the two boards is facilitated by a 6‐pin interface: four SPI lines (MOSI, MISO, SCK, CS), ground, and 3.3 V power (Figure 7c).

The system runs on FreeRTOS, where a high‐priority task (“captureTask”) samples data from all six channels at 250 Hz using vTaskDelayUntil to ensure timing consistency. Each sample is stored as a pack in a FIFO queue. Once eight packs are accumulated, they are transmitted via BLE UART in a text‐based format for ease of visualization and processing. Each value is scaled to a range of 000–999 (with clamping above 1024, which did not occur during testing), and separated by commas or newlines. The resulting data rate is 6000 bytes s^−1^ (4 bytes × 6 channels × 250 samples s^−1^). Data is received on iOS and Android devices using the open‐source Bluefruit Connect app by Adafruit, which supports UART monitoring, real‐time plotting, and CSV export for post‐analysis.^[^ ^68^ ^]^ The complete microcontroller firmware for the Arduino platform is now available as open‐source material on the Zenodo platform. The link to the code is in Note S10 (Supporting Information).

### 2.5 Assessing JBM Using the Fabrigami TENG Sensor

**Figure 8:**
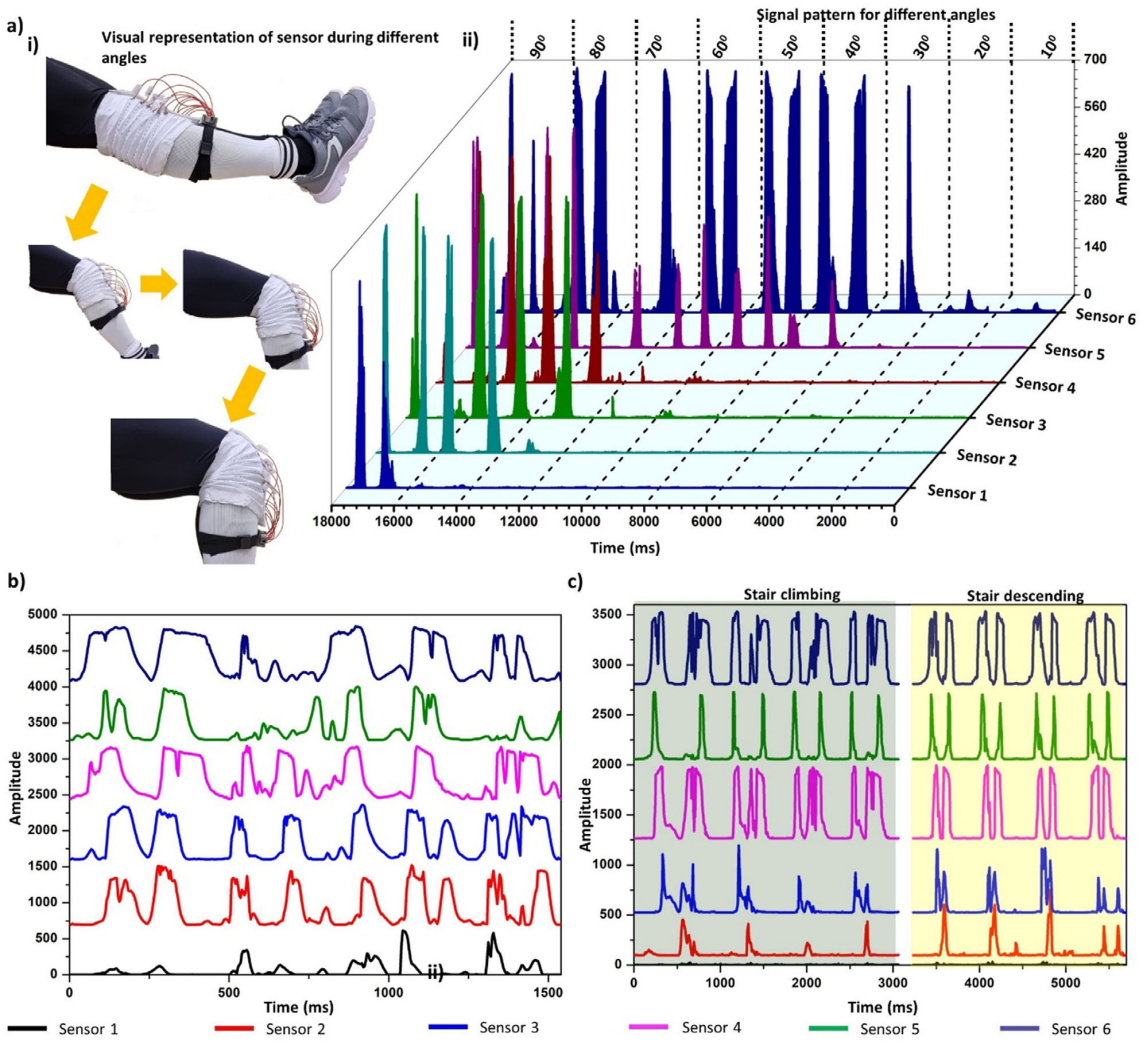
Application development by evaluating sensor sensitivity performance: a‐i) visual representation of knee bending, and (ii) sensors response at different bending angles. Sensor measurements during b) running, c) climbing and descending stairs.

The primary objective of the developed sensor is to detect the JBM of the knee. In daily practice, runners and athletes often experience variations in their JBM due to changes in body posture or the initiation and conclusion of joint movements during various exercises.^[^ ^69^ ^]^ We employed six sensors to detect angles ranging from 10° to 90° with 10° intervals. This represents the first development of a sensor using the TENG concept combined with a fabrigami structure. Once the device was assembled, it was attached to the knee, as shown in **Figure** **8**a, where the fabrigami TENGs are positioned posterior to the knee joint and signal detection was performed at different angles. We used a commercial angle detection sensor (Bend Labs 1‐Axis Flex Sensor Evaluation Kit) to monitor the actual knee angle, and the responses of the various sensors at different angles are depicted in Figure 8a‐ii. Notably, up to 30°, there is a distinct change in pressure sensitivity for Sensor 6, and beyond this angle, all sensors are activated to provide different signal patterns corresponding to various angles. A demonstration of this setup is provided in Video S2 (Supporting Information), showing the whole operation sequence: i) sensor positioning around the knee joint, ii) actuation during flexion–extension cycles, and iii) immediate signal display on the mobile interface. This real‐time operation confirms the system's capability to function as a wearable JBM monitoring sensor without noticeable latency in data acquisition or transmission.

Additionally, we tracked fast movements such as running (Figure 8b), climbing and descending stairs (Figure 8c) to get an overall idea about the sensor's limitations. It was evident that the sensor can successfully detect slow and fast movements accurately, providing real‐time angle measurements with temporal information to determine the time taken to reach a set angle allowing analysis of the wearer's performance of repeated exercises. This allows the identification of the knee angle and movement frequency throughout the activity.

**Figure 9:**
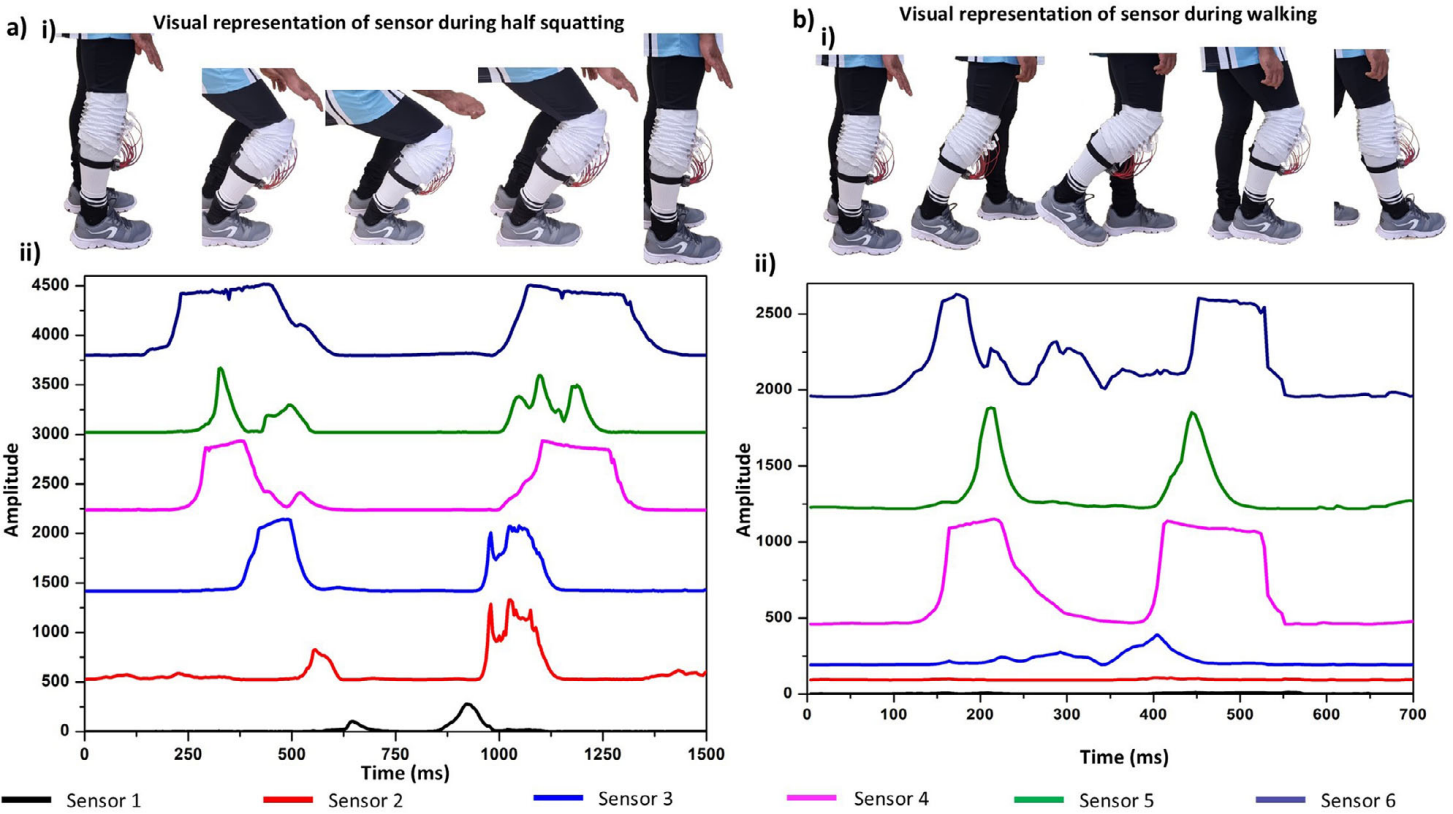
Further clarification on the limitations of the sensor: a) visual representation (i) and signal pattern (ii) during half squatting, b) visual representation (i) and signal pattern (ii) during walking.

To further understand the signal patterns, we conducted half‐squat exercises, i.e., targeting the top half range of motion during a squat (**Figure** **9**a) and walking (Figure 9b). During the squatting process, we can see that the sensors are activated in a characteristic pattern as the knee bends and the folds are compressed. As the knee bends, each sensor responds in sequence after the topmost sensor, i.e., sensor 6, is activated, in correspondence with the knee flexion, which increases the knee flexion angle, e.g. sensor 5 has a 92 ms time delay after sensor 1, corresponding to bending by 30–40°. Combining these results, we can gain insight into different exercises and movement patterns. The developed sensor proved particularly effective in monitoring exercises like squatting, offering detailed insights into knee movement. Moreover, the activation of sensor 1 when the person reaches the bottom position during squatting indicates the knee angle reaching up to 90°.

Walking is composed of two primary phases: the stance phase and the swing phase. As depicted in Figure 9b‐i, key moments include standing, initiating a left step while preparing for right heel‐off, right leg swing forward, and right toe‐down.^[^ ^70^, ^71^ ^]^ In this experiment, the sensor was attached to the right knee, and the corresponding signal profile was recorded, as shown in Figure 9b‐ii. The knee demonstrates a characteristic flexion‐extension pattern during the gait cycle, with peak flexion occurring during the initial swing phase, followed by extension before foot contact. Lower flexion angles are typically maintained during most of the stance phase. The sensor amplitude shown in the graph corresponds to these angle changes, with higher amplitude signals correlating with increased knee flexion. Although the sensor did not fully distinguish between the stance and swing phases, it successfully captured the maximum knee flexion angle during the gait cycle. As a preliminary step toward future energy‐harvesting applications of the fabrigami sensor, we integrated the sensor into the fabrigami structure and successfully charged a capacitor to 2 V within 76 s. The stored energy was then used to power a digital watch for approximately 10.5 s (Video S3, Supporting Information). These results offer promising insights into providing a more detailed understanding of knee JBM. With future improvements, this sensor could evolve into a self‐powered, sustainable tool for monitoring exercise and therapeutic performance for sports and health.

## 3 Conclusion

In this study, we have developed a pioneering TENG sensor that integrates a fabrigami structure with advanced electrospun materials for enhanced knee JBM detection. This work represents a significant advancement in wearable sensing and self‐powered devices, addressing both the technological and practical challenges associated with these applications.

Incorporating Ag nanoparticles into CA has yielded a notable change in the surface structure and an enhancement in triboelectric performance, as confirmed by comprehensive material characterization techniques, including SEM, FTIR, and XRD. These findings underline the successful modification of the CA matrix to improve its triboelectric properties. Concurrently, the PVDF fibers exhibited a high β‐phase content, essential for optimal piezoelectric performance. The mechanical and wettability properties of the electrospun materials were also significantly influenced by the doping process, which had implications for their functionality in TENG applications.

The TENG device demonstrated exceptional self‐powered capabilities, with a maximum *V*_OC_ of 155.9 V, a *J*_SC_ of 8.134 mA m^−2^, and a *Q*_SC_ of 65.622 µC m^−2^ achieved with 1.5% Ag‐CA. This performance is among the highest reported for similar devices and indicates the successful optimization of the material composition and structure. The power density of 0.029 W m^−2^, along with practical demonstrations such as LED lighting and capacitor charging, showcases the device's potential for real‐world applications, particularly in powering low‐energy electronics. The power conversion efficiency of the fabrigami TENG sensor was estimated in the range of ≈4.6–92.8%, depending on the elastic compression of the electrospun layers. These values confirm that the device can operate efficiently under realistic conditions while primarily serving as a robust self‐powered sensor for joint biomechanics monitoring.

The developed sensor offers high sensitivity and accuracy in detecting knee JBM, with peak‐to‐peak voltages responding proportionally to applied forces and rapid response times. The sensor's performance across various movements, including walking, stair climbing, and squatting, demonstrates its effectiveness in dynamic monitoring scenarios. This capability is crucial for applications in sports and rehabilitation, providing detailed insights into joint movement and facilitating advanced wearable technologies. By further enhancing the sensor's detection range and integrating advanced signal processing techniques, there is significant potential to improve both its sensitivity and accuracy.

The successful design and fabrication of the fabrigami structure, using thermal imprinting techniques, emphasizes the innovative approach taken in this study. The resulting 3D structure not only enhances the TENG's operational efficiency through optimized contact‐separation cycles but also exemplifies the practical integration of flexible materials with advanced fabrication methods.

It should be noted that the primary function of the fabrigami device is as a sensitive wearable sensor for joint biomechanics monitoring, rather than as a dedicated energy‐harvesting system. Consequently, device design choices including Ag nanoparticle concentration, origami structure, and testing conditions were optimized for mechanical flexibility and sensing stability rather than maximum electrical power output. The reported power density values serve as an initial proof‐of‐concept demonstration of the device's potential for future self‐powered applications. Comprehensive impedance matching, load optimization, and power enhancement strategies will be explored in dedicated future studies.

As summarized in Note S11 (Supporting Information), fabrigami‐based sensors achieve a unique balance of high conformability, mechanical stability, and tunable sensitivity. Compared with other origami or kirigami structures that focus on peak sensitivity or extreme durability, fabrigami's fabric folds accommodate multidirectional strain while maintaining stable triboelectric output during repetitive knee flexion. This combination of programmability, comfort, and self‐powered sensing capability underscores its strong potential for scalable deployment in joint biomechanics monitoring.

In conclusion, this research presents a groundbreaking approach to the integration of triboelectric materials with a fabrigami structure, achieving significant advancements in JBM sensing. The results highlight the transformative potential of these innovations for wearable technology and self‐powered systems, marking a substantial contribution to the field of advanced materials.

## 4 Experimental Section

### PVDF Electrospinning

PVDF with molecular weight 530 000 (Sigma‐Aldrich, 347078), acetone (ACS reagent, ≥ 99.5% Sigma‐Aldrich, 179124) and N,N‐dimethylformamide(DMF) (HPLC, ≥ 99.9% Sigma‐Aldrich, 570547) were used to prepare PVDF electrospinning precursor. 20 wt% PVDF was put in DMF:acetone (3:2) solution and magnetically stirred for 4 h at 50 °C on a hot plate. Once the solution reached room temperature, it was loaded into a syringe with a 23 G blunt needle. Electrospinning was conducted at room temperature (20± 2 °C) and relative humidity of 50± 5% (no specific measures were taken to control the environmental conditions) using a KDS200P syringe pump with a bespoke rotary collector. The flow rate of 2 mL h^−1^, tip‐to‐collector distance of 15 cm and applied voltage of 18 kV was maintained during the process. PVDF mat was collected onto aluminum foil at 1500 rpm. After collection, the mat was carefully removed from the aluminum foil, dried inside a fume hood for 48 h, and dried in an oven at 80 °C for 1 h to confirm the complete removal of DMF and Acetone residues.

### Ag‐CA Electrospinning

CA with molecular weight 30 000 (Sigma‐Aldrich, 180955) and Ag nanoparticles ink (50 wt%, dispersion in tripropylene glycol monomethyl ether, Sigma‐Aldrich, 796042) were used to prepare Ag‐doped cellulose acetate precursor. 20 wt% CA was mixed in DMF:acetone (2:3) solution and magnetically stirred for 4 h at room temperature. A series of solutions (0%, 0.5%, 1%, 1.5%, 2%, 2.5% Ag wt/wt in CA precursor) were prepared to add Ag nanoparticle solution and stirred for 1 h for a homogenous solution. The precursor was loaded into a syringe with a 21 G blunt needle, and electrospinning was carried out with a flow rate of 2 mL /h^−1^, tip‐to‐collector distance of 15 cm, applied voltage of 15 kV and 1500 rpm under similar room temperature and humidity conditions which was applied for PVDF. Ag‐CA mat was collected onto aluminum foil and dried at room temperature for 48 h. After that, samples were further dried in an oven at 50 °C for a 4 h.

### Material Characterization

EVO LS15 (Zeiss) was used for SEM imaging and EDS initial analysis. High magnification imaging and EDS color mapping for Ag‐CA and PVDF was carried out using the Hitachi s500 field emission SEM. FTIR was carried out using PerkinElmer Spectrum two FT‐IR. XRD analysis was carried out with Bruker D5000.

XPS analysis was performed on a Scienta Omicron XPS system with Argus CU multiplate detector and a monochromated Al K‐alpha X‐ray source with an excitation energy of 1486.6 eV. Due to charging during scanning because of the insulating nature of the CA samples, all spectra were shifted such that the maximum intensity of the C‐C component of the C1s peak appeared at 284.5 eV.

KPFM measurements were conducted using an MFP‐3D atomic force microscope (Oxford Instruments) with conductive PPP‐EFM probes (nanosensors) having an tip radius of ≈ 20 nm and a resonance frequency of 75 kHz. Measurements were carried out in lift mode, whereby the topography was first recorded in amplitude modulation mode (widely acceptable method in literature),^[^ ^72^, ^73^, ^74^ ^]^ followed by lifting the tip by 50 nm to measure the CPD. KPFM operates through a feedback mechanism, whereby an external bias is applied to minimize the CPD between the tip and the sample during the lift pass. The measurements were carried out under optimized conditions with consistent parameters across all samples (50 nm lift height, 0.5 Hz scan rate). The tip calibration was carried out using a standard aluminum and gold sample (Bruker). To ensure the tip did not deteriorate, the standard sample was measured both before and after the actual sample measurements (Note S12, Supporting Information). All KPFM measurements were recorded under normal ambient conditions.

Tensile properties were characterized by a Zwick Z005 tensile tester, and the wetting angle was analyzed using the FTA200 dynamic contact angle analyzer.

### Triboelectric Characterization

Initially, the samples were characterized based on the outputs: *V*_OC_, *J*_SC_ and *Q*_SC_. Electrospun samples were cut into 40 × 40 mm and attached to a copper electrode using 3 M 9713XYZ conductive double tape. After that, both electrodes were attached to insulators, as shown in Note S5 (Supporting Information). TENG contact separation mode characterization technique was used with a bespoke setup for initial characterizations. The applied force was measured using a force gauge (to maintain constant force), and *V*_OC_ was measured using a Tektronix TBS 1052B‐EDU digital oscilloscope. Short circuit current and charge were measured using a Keithley 6517B electrometer, and data was recorded using a Py‐visa based Python script for further processing.

## Conflict of Interest

K.R.S.D.G., Z.F., G.B.M., and S.M.C. are the inventors of the patent application with the title “A Fabrigami Sensor” under UK Patent Application No. 2505562.5. The other authors do not declare conflicts of interest.

## Supporting information



Supporting Information

Supplemental Video 1

Supplemental Video 2

Supplemental Video 3

## Data Availability

The data that support the findings of this study are available from the corresponding authors upon reasonable request.
