# Supplementary material for: Innovative Self‐Powered Sensing: Potential of Fabrigami and Electrospun Nanofiber‐Based Triboelectric Nanogenerator for Joint Biomechanics Monitoring
Source: Small. 2025 Sep 27;21(45):e06363. doi: 10.1002/smll.202506363 (PMC12614136; doi:10.1002/smll.202506363)
Supplement: Supplementary file 1 — Supporting Information [file SMLL-21-e06363-s002.docx]

Supplementary information for

**Innovative Self-Powered Sensing: Potential of Fabrigami and Electrospun Nanofiber-Based Triboelectric Nanogenerator for Joint Biomechanics Monitoring**

*K. R. Sanjaya D. Gunawardhana ^1, 2, *^, Zhou Fang^1^, Garrett B. McGuinness ^3^, Luz Alejandra Magre Colorado^1^, Sonal Santosh Baberwal^1^, Waseem Ahmad Wani^4^, Brian J. Rodriguez^4^, Robert O'Connor^5^, Ciara Smullen^5^, Tomás E. Ward ^2,6^, Shirley M. Coyle ^1, 2, *^*

*^1^School of Electronic Engineering, Dublin City University, Glasnevin, Dublin 9, Ireland*

*^2^Insight Research Ireland Centre for Data Analytics, Dublin City University, Glasnevin, Dublin 9, Ireland*

*^3^School of Mechanical Engineering, Dublin City University, Glasnevin, Dublin 9, Ireland*

*^4^School of Physics and Conway Institute, University College Dublin, Belfield, Dublin 4,*

*Ireland*

*^5^School of Physical Sciences, Dublin City University, Glasnevin, Dublin 9, Ireland*

*^6^School of Computing, Dublin City University, Glasnevin, Dublin 9, Ireland*

**Corresponding authors –* [*Sanjaya.gunawardhana2@mail.dcu.ie*](mailto:Sanjaya.gunawardhana2@mail.dcu.ie)*;* [*Shirley.coyle@dcu.ie*](mailto:Shirley.coyle@dcu.ie)

**Supplementary Note 1**

**Comparission of previous electrospun triboelectric self powered sensor performance.**

For comparing the sensor performance with previously published results (Table S1) we did a literature surway using following criteria.

Material fabrication – at least one triboelectric layer is modified via electrospinning

Applicaton – Wearable signal monitoring or related pressure sensitivty

Pressure sensitivty – V kPa^-1^

Table S1: Summarization of previously developed electrospun layer based wearable sensors.

| **Year** | **Materials** | **Energy harvesting performance** | **Self-powered sensing performance** | **Application** |
| --- | --- | --- | --- | --- |
| 2018[1] | Ag nanoparticles ink – tribopositive PVDF nanofibers – tribonegative | V_OC_ $\sim$ 25 V  I_SC_ $\sim$ 320 nA  Power $\sim$ 0.12 mW  Stable for 1800 cycles | 0.385 V kPa^-1^  Approximately 0-40 kPa range | Identification of breathing patterns normal and rapid. |
| 2018[2] | PDMS core-shell PDMS ion gel / PVDF-HFP nanofiber – tribonegative Kapton film – tribopositive | V_OC_ $\sim$ 75 V  I_SC_ $\sim$ 10 μA cm^-2^  Power $\sim$ 0.9 W m^-2^  Stable after 6 months of shelf life. | 0.43 V kPa^-1^  0-1.6 kPa  0.068 V kPa^-1^  0-700 kPa | NA |
| 2019[3] | PVDF/Ag NW, nanofibers- tribonegative ethyl cellulose nanofibers- tribopositive | V_OC_ -62.73 V  I_SC_ – 490.7 nA  Q_SC_ – 22.35 nC power density – 7.2 mW m^-2^  Stable for 7200 cycles | 1.67 V·kPa^–1^  0–3 kPa  0.20 V·kPa^–1^  3–32 kPa | Joint movement detection (elbow, knee ancles) |
| 2020[4] | PLGA/AgNW/PVA facial electrospun layer – tribonegative  PTFE – tribopositive-  Middle sandwiched AgNW work as the electrode for tribonegative layer. | V_OC_ $\sim$90 V  I_SC_ $\sim$ 2.5 μA  Q_SC_ $\sim$30 nC power density - 130 mW m^-2^ through 500 mΩ  Stable signal for 50000 cycles. | 0.011 V kPa^-1^  0-40 kPa | Frown movements, eye blinking, breathing behaviour monitoring |
| 2020[5] | PVDF nanofibers – tribonegative human skin – tribopositive | V_OC_ $\sim$30 V | 0.18 V kPa^-1^ 0-175 kPa | Curvature or bending of the wrist |
| 2021[6] | TiO_2_@PAN electrospun coated with PTFE – tribonegative  Nylon film – tribopositive | V_OC_ $\sim58$ V  I_SC_ $\sim$ 100nA  Q_SC_ $\sim25$ nC  Power density -48.6 mW m^–2^ through 4160 MΩ  Stable for 3500 cycles. | 5.2 V kPa^–1^  0–4 kPa  0.6 V kPa^–1^  >4 kPa | Distinguish human motions such as walking, running, squatting, and skipping |
| 2021[7] | Single electrode mode TPU electrospun – tribonegative layer  Ag NW on PVA/CS electrospun layer as electrode | V_OC_ $\sim12$ V  I_SC_ $\sim$ 200 nA  Q_SC_ $\sim5$ nC stable for 1200 cycles. | 0.3086 V kPa^–1^  6.65–19.21 kPa. | Impact of volleyball on skin during practice. |
| 2021[8] | poly-DADMAC/nylon-11 nanofiber mat) – tribopositive  PVDF-TrFE nanofiber mat – tribonegative | V_OC_ - 380 V  I_SC_ - 80 μA  Q_SC_ – 200 nC power density - 7.6 W m^-2^  Through 4 MΩ  Dielectric constant – 10 at 33 wt% poly-DADMAC  Stable for 86000 cycles | 1.01 V kPa^-1^  0-16 kPa  0.355 V kPa^-1^  16-36 kPa | Distinguish walking patterns (slow, fast jogging etc) |
| 2022[9] | Core sheath yarn electrospinning  PCL -tribopositive  PVDF/PTFE – tribonegative | V_OC_ - 20 V  I_SC_ – 2.26 μA  Q_SC_ – 6.3 nC power density – 2.2 mW m^-2^  Through 20 MΩ  Stable for 10000 cycles. | 0.367 V kPa^-1^  0-35 kPa range | Lying and standing up can be distinguished |
| 2022[10] | Co-NPC/PVDF composite NFs – tribonegative layer  Nylon 11 nanofibres – tribopositive layer | V_OC_ – 710 V  J_SC_ – 210.96 mA m^-2^  Charge density – 392 μC m^-2^ power density – 19.24 W m^-2^  Through 5 MΩ  Stable for 60000+ cycles  Dielectric constant 22 for Co-NPC/PVDF composite NF  Power conversion efficiency – 58.3% | 6.39 V kPa^-1^  1.5-16 kPa  0.36 V kPa^-1^  16-65 kPa | Walking, slow running, jumping, fall down can be distinguished |
| 2022[11] | Nylon 66 electrospun – tribopositive  Siloxane – PVDF -tribonegative | V_OC_ – 645.65 V  I_SC_ – 276 μA  Q_SC_ – 212 nC power density – 13.25W m^-2^  Through 1 MΩ  Dielectric constant 36.25 at 20% siloxane in PVDF  Stable for 60000 cycles. | 12.062 V kPa^-1^  0-3 kPa  2.58 V kPa^-1^  3-25 kPa | Dynamic pressure can be measured on a wearable application |
| 2023[12] | TPU electrospun – tribopositive  Ecoflex film - tribonegative | V_OC_ – 123 V  I_SC_ – 1.7 μA  Q_SC_ – 41.5 nC power density – 122 mW m^−2^  Through 500 MΩ  Stable for 3000 cycles. | 15.94 V kPa^-1^  0-3 kPa  3.70 V kPa^-1^  3-15.63 kPa | Detecting pulses at different sites (the radial, superficial temporal, and carotid artery) |
| 2023[13] | CNT incorporated CA electrospun – tribopositive  PVDF electrospun -tribonegative | V_OC_ $\sim$ 112.8 V  I_SC_ $\sim$ 29.9 μA  Q_SC_ – 35.5 nC power density – 0.74 W m^−2^  Through 80 MΩ  Stable for 5000 cycles | 3.03 V kPa^-1^  0-6.8 kPa  0.11 V kPa^-1^  6.8-65 kPa | Finger, wrist and arm bending angle was measured using this device |
| 2024[14] | polarized polyvinylidene fluoride-barium titanate electrospun – tribonegative  Ni fabric - tribonegative | V_OC_ $\sim$ 240 V  I_SC_ $\sim$ 11 μA  Q_SC_ – 80 nC power density – 4.62 W m^−2^  Through 50 MΩ  Stable after 30 days and 3500 cycles. | 2.62 V kPa^-1^  1-10 kPa | Getting - 30°, 60°, 90° degrees of knee, elbow and finger |
| 2024[15] | PEO@Poly-DADMAC electrospun composite – tribopositive  PVDF electrospun - tribonegative | V_OC_ $\sim$ 980 V  I_SC_ $\sim$ 30 μA  Q_SC_ – 145 nC power density – 5.6 W m^−2^  Through 10 MΩ  Dielectric constant – 8 at Poly-DADMAC 1.25%  Stable for 20000 cycles. | 8.923 V kPa^-1^  1-25 kPa  2.265 V kPa^-1^  25-87 kPa | Differentiate walking, running, jumping, cycling (both straight and with bending shoulders), sitting, and standing up |
| 2025 (our work) | Ag-CA electrospun -tribopositive  PVDF electrospun – tribonegative | V_OC_ -155.9 V  J_SC_ – 8.134 mA m^-2^  Q_SC_ – 65.622 nC power density – 0.029 W m^-2^  Stable for 15000 cycles and after 8 months of shelf life.  Power conversion efficiency of  4.6–92.8% for 100–5 µm elastic compression | 11.667 V kPa^-1^  0-6.25 kPa  5.157 V kPa^-1^  6.25-18.75 kPa | Detecting knee biomechanics targeting rehabilitation and exercise (running, walking, squatting etc) |


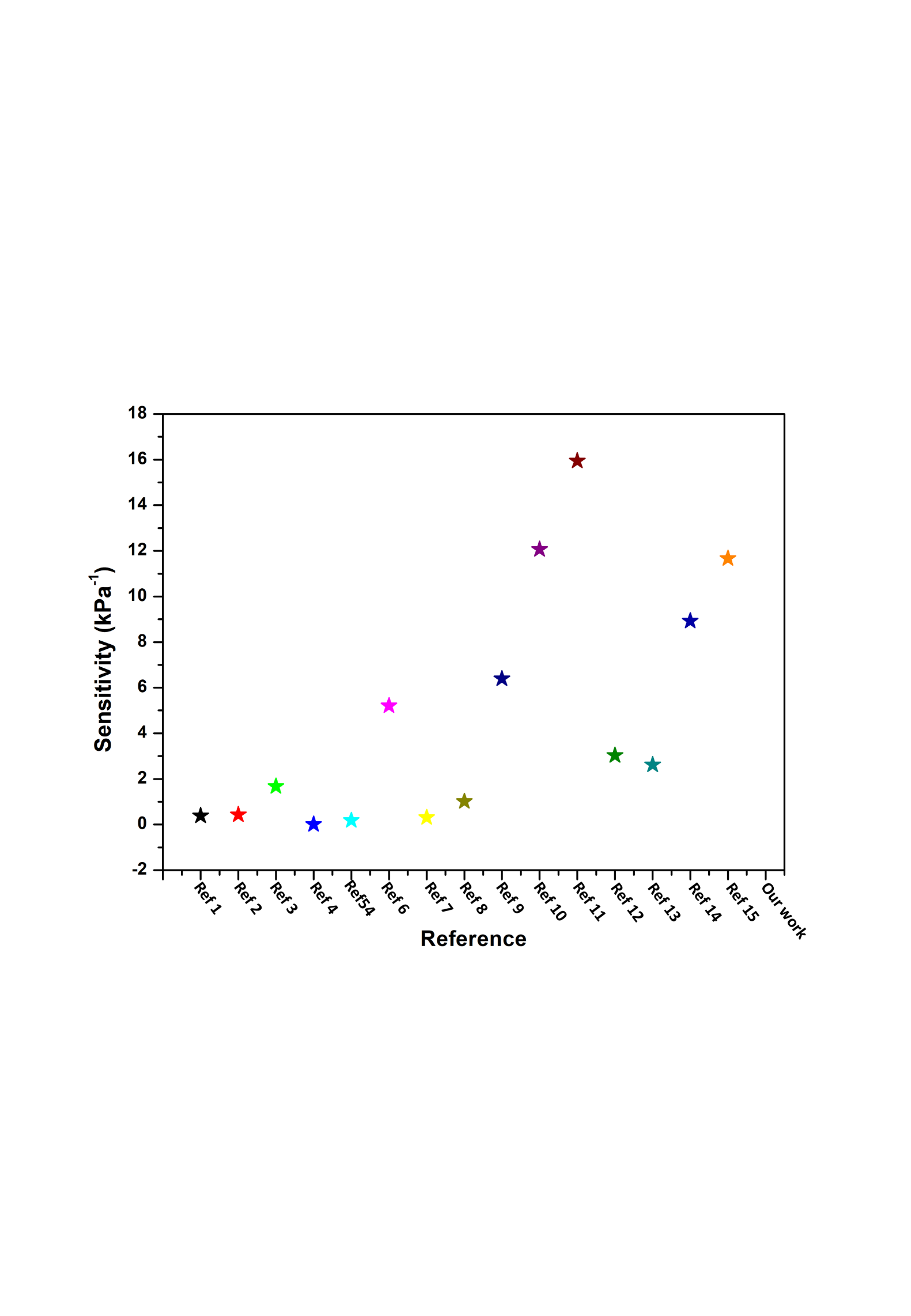


Figure S1: Comparison of sensitivity of different literature results with newly developed sensor.

**Supplementary Note 2**


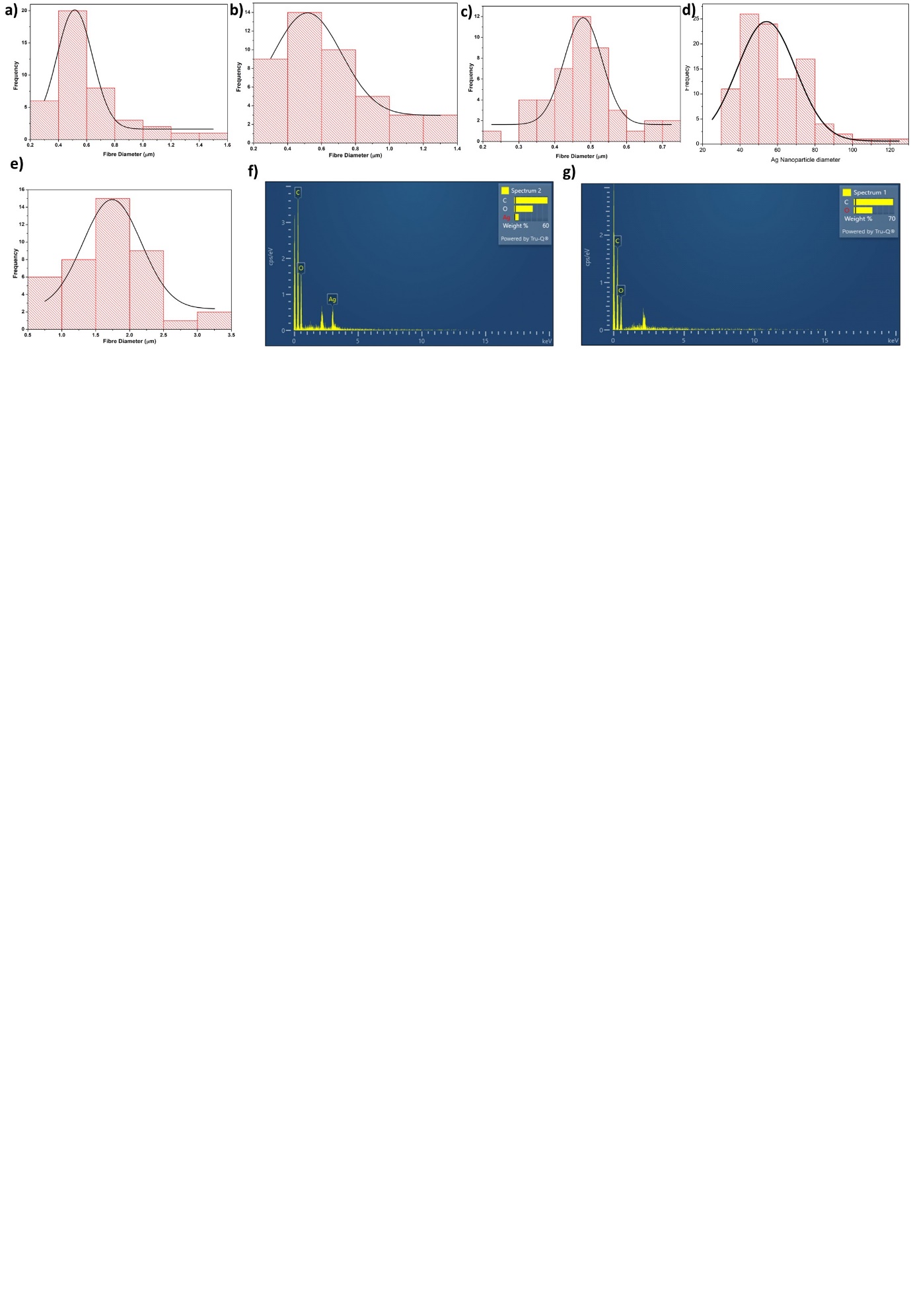
 **Fibre diameter distribution and EDS analysis of CA, Ag-CA and PVDF.**

Figure S2: Fibre diameter distribution of a) CA only, b) 1.5% Ag in CA, c) 2.5% Ag in CA,d) Ag nanoparticles, and d) PVDF., EDX on a selected point in e)pure CA and f)1.5% Ag in CA.

**
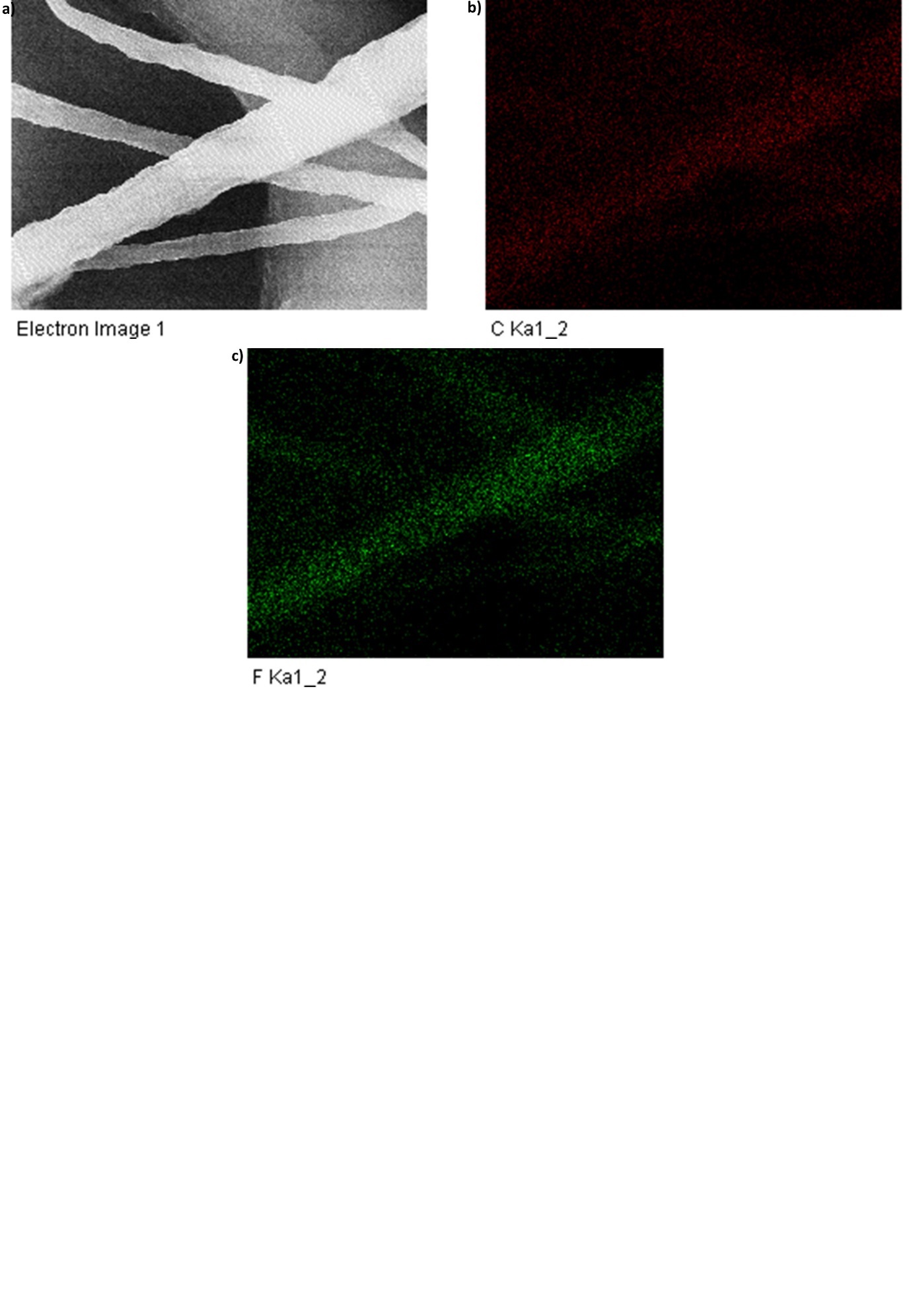
**

Figure S3: EDS analysis results of PVDF nanofibers. a) SEM image use for EDS analysis, Elemental map of b) Carbon and c) Fluorine on PVDF micro-nanofibers.

**Supplementary note 3**

**Β phase calculation for PVDF sample**

Lambert-Beer law to calculate the β phase of PVDF electrospun layer[16].

$F\left( \beta\right)= \frac{A_{\beta}}{\left( \frac{K_{\beta}}{K_{\alpha}} \right)A_{\alpha}+A_{\beta}}$ (SE 1)

Where K_β_ = 7.7 $\times$ 10^4^ and K_α_ = 6.1 $\times$ 10^4^ for coefficient of absorbance at 840 cm^-1^ and 762 cm^-1^, respectively. A­_β_ and Aα Using the FTIR absorption results we found that,

| A_β_ | 0.071 |
| --- | --- |
| A_α_ | 0.018 |

Using these parameters final value calculated F(β) = 75.75 %

**Supplementary Note 4**

**Theoretical simulation of Ag-CA and PVDF with DDEF model**

To further understand the charge transfer mechanism between the PVDF and CA a developed theoretical model was used. There have been some promising theoretical models known as V-Q-X relationship, parallel plate capacitor model and DDEF model. This DDEF model accounts for the electric field variations between oppositely charged finite surfaces as they undergo contact-separation movements, offering a more comprehensive explanation of TENG operation. Among these models DDEF was selected for this experiment due to the higher accuracy regarding the prediction of V_OC_, Q_SC_ and J_SC_[17].

Simulation starts with approximating the average electric field of above a specific surface with length L and width W above its midpoint along an axis perpendicular to the surface ($E_{x}$). Considering our experiment L=W=4 cm, surface charge density $\sigma$ which is place in a medium with permittivity $\varepsilon$ the simplified equation as given in SE 2, (original equation can be found in ref[18], [19])

$E_{x}=\frac{\sigma}{\pi\varepsilon}\arctan\left( \frac{4\times{10}^{-4}}{x\sqrt{x^{2}+8\times{10}^{-4}}} \right)=\frac{\sigma}{\pi\varepsilon}f\left( x \right)$ (SE 2)

where $x$ is the perpendicular distance in consideration from the charged surface.

Considering equal positive and negative triboelectric charge of $\sigma_{T}$ is uniformly distributed at the surface of PVDF and CA substrates and considering electrodes are in open circuit configuration (the charge on the electrode $\sigma_{U}=0$) we can calculate the overall electric field at the PVDF and attached electrode connecting interface using SE 3.

$E_{Total, PVDF/electrode}=\frac{\sigma_{T}}{\pi\varepsilon_{1}}((f\left( d_{1} \right)-f\left( x_{0}+d_{1} \right))$ (SE 3)

Where, $\varepsilon_{1}$ and d_1_ are permittivity and the thickness of PVDF, respectively. $x_{0}$ is the separation between PVDF and Ag-CA layer (Figure S4).


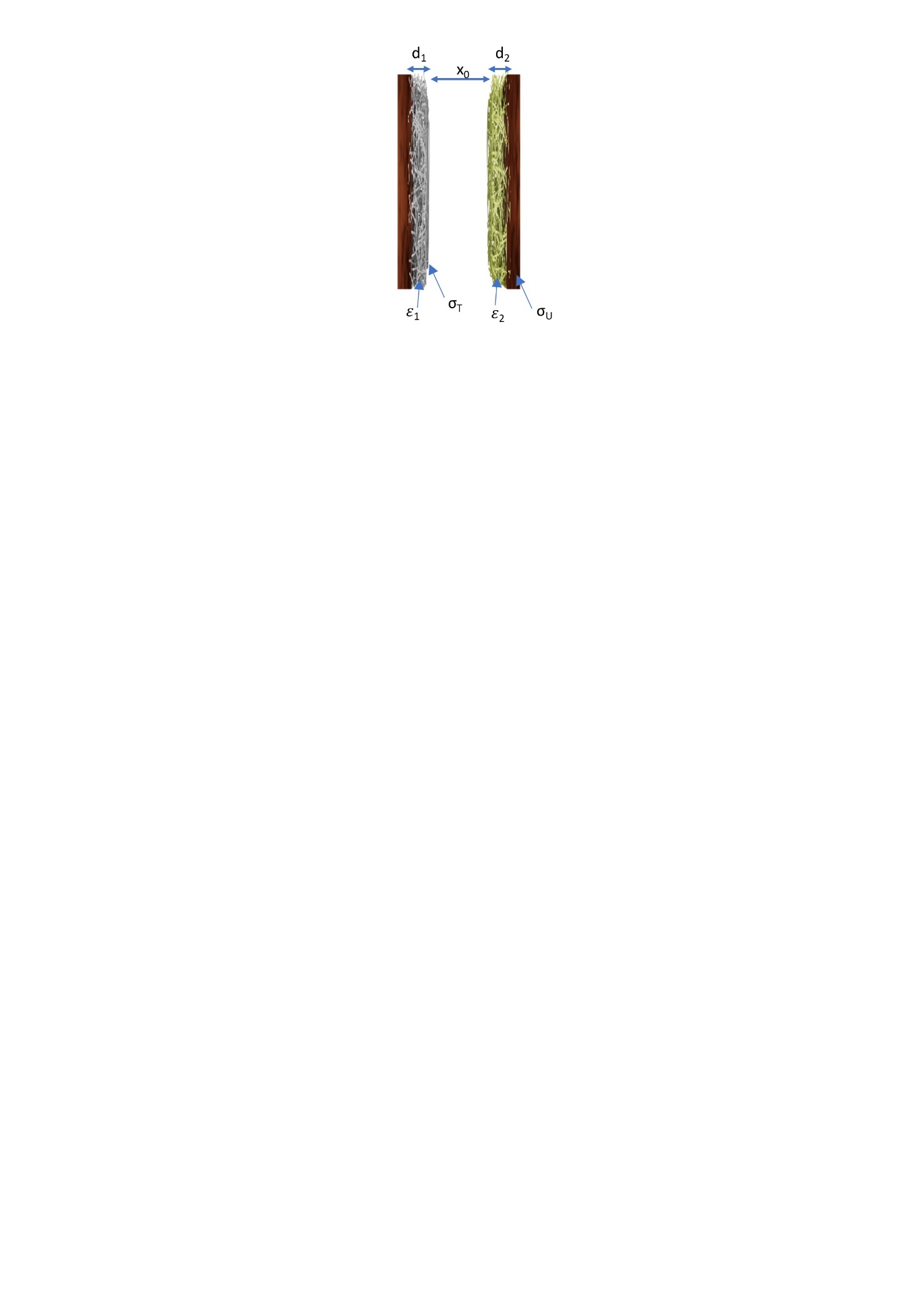


Figure S4: Representation of parameters for theoretical calculation.

Electric potential on the PVDF and attached electrode interface (V_1_) can be calculated by integrating SE 3 as shown in SE 4

$V_{1}=\frac{\sigma_{T}}{\pi\varepsilon_{1}}\left( \int_{d_{1}}^{d_{1}+x_{0}} f\left( x \right)dx \right)=\frac{\sigma_{T}}{\pi\varepsilon_{1}}\left[ Y(x) \right]_{d_{1}}^{d_{1}+x_{0}}$ (SE 4)

Considering the analytical solution for the integration of f(x), Y(x) has been developed taking the solution from ref[19] and modifying this with our parameters to generate the following SE 5 equation.

$Y\left( x \right)=x arctan\left( \frac{4\times{10}^{-4}}{x\sqrt{x^{2}+8\times{10}^{-4}}} \right)-2\times{10}^{-2}ln\left( \frac{\sqrt{x^{2}+8\times{10}^{-4}}+2\times{10}^{-2}}{\sqrt{x^{2}+8\times{10}^{-4}}-2\times{10}^{-2}} \right)$ (SE 5)

Considering all parameters and taking into account Y(x) we can now calculates the V_OC_ as given in SE 6,

$V_{OC}=\frac{\sigma_{T}}{\pi}\left( \frac{1}{\varepsilon_{1}}\left[ Y(x) \right]_{d_{1}}^{d_{1}+x_{0}}-\frac{1}{\varepsilon_{2}}\left[ Y(x) \right]_{d_{2}}^{d_{2}+x_{0}} \right)$ (SE 6)

Where $\varepsilon_{2}$ and d_2_ are permittivity and the thickness of Ag-CA, respectively.

Considering the short circuit condition, we can calculate J_SC_ using V_1_ and V_2_ equations. When the attached electrodes are in short circuit condition V_1_ -V_2_ =0 due to free charges which can move from one electrode to other electrode to neutralize any potential difference. At equilibrium state the potential at the PVDF attached electrode interface can be written as SE 7.

$V_{1}=\frac{\sigma_{T}}{\pi\varepsilon_{1}}\left( \int_{d_{1}}^{d_{1}+x_{0}} f\left( x \right)dx \right)-\frac{\sigma_{U}}{\pi\varepsilon_{1}}\left( \int_{0}^{{d_{1}+d}_{2}+x_{0}} f\left( x \right)dx \right)$ (SE 7)

Taking the same conditions at Ag-CA and attached electrode, interface equations can be derived for V2 and finally considering an open circuit condition we can get the equations for $\sigma_{U}$ and J_SC_ as given in SE 8, SE 9 respectively.

$\sigma_{U}=\sigma_{T}\frac{\left( \frac{1}{\varepsilon_{1}}\left[ Y(x) \right]_{d_{1}}^{d_{1}+x_{0}}+\frac{1}{\varepsilon_{2}}\left[ Y(x) \right]_{d_{2}}^{d_{2}+x_{0}} \right)}{\left( \frac{1}{\varepsilon_{1}}+\frac{1}{\varepsilon_{2}} \right)\left[ Y(x) \right]_{0}^{d_{1}+d_{2}+x_{0}}}$ (SE 8)

$J_{SC}=\frac{d\sigma_{U}}{dt}$ (SE 9)

Selecting a contact and separation profile these equations can be used to simulate results for the PVDF/Ag-CA TENG development.

Parameters used for simulation.

For the PVDF tribonegative layer, the low-frequency permittivity is well documented: $\varepsilon_{r}$ values typically range between 8–12 at ~1 kHz, with datasheets reporting $\varepsilon_{r}$~4.5–9.5 depending on crystallinity and frequency. Peer-reviewed studies consistently confirm ≈ 10–12 for dense PVDF films. On this basis, we adopted $\varepsilon_{r}$ = 10 as a conservative, literature-grounded value[20], [21].

For the Ag–CA tribopositive layer, neat CA films are generally reported with ε_r_ values of 3.5–7.5 at ~1 kHz. Electrospun mats show lower effective $\varepsilon_{r}$due to porosity. At the low Ag loadings used here (≈1.5 wt%), only a modest increase is expected, far below the percolation threshold. Accordingly, we set $\varepsilon_{r}$ (Ag–CA) = 5.0, consistent with reported values for CA and dilute composite systems [22], [23], [24], [25]

Measured thickness of the PVDF $\sim$ 57 μm

Measured thickness of Ag-CA $\sim$ 55 μm

Experimental charge density matched with DDEF prediction at σT =66.4 µC m^-2^


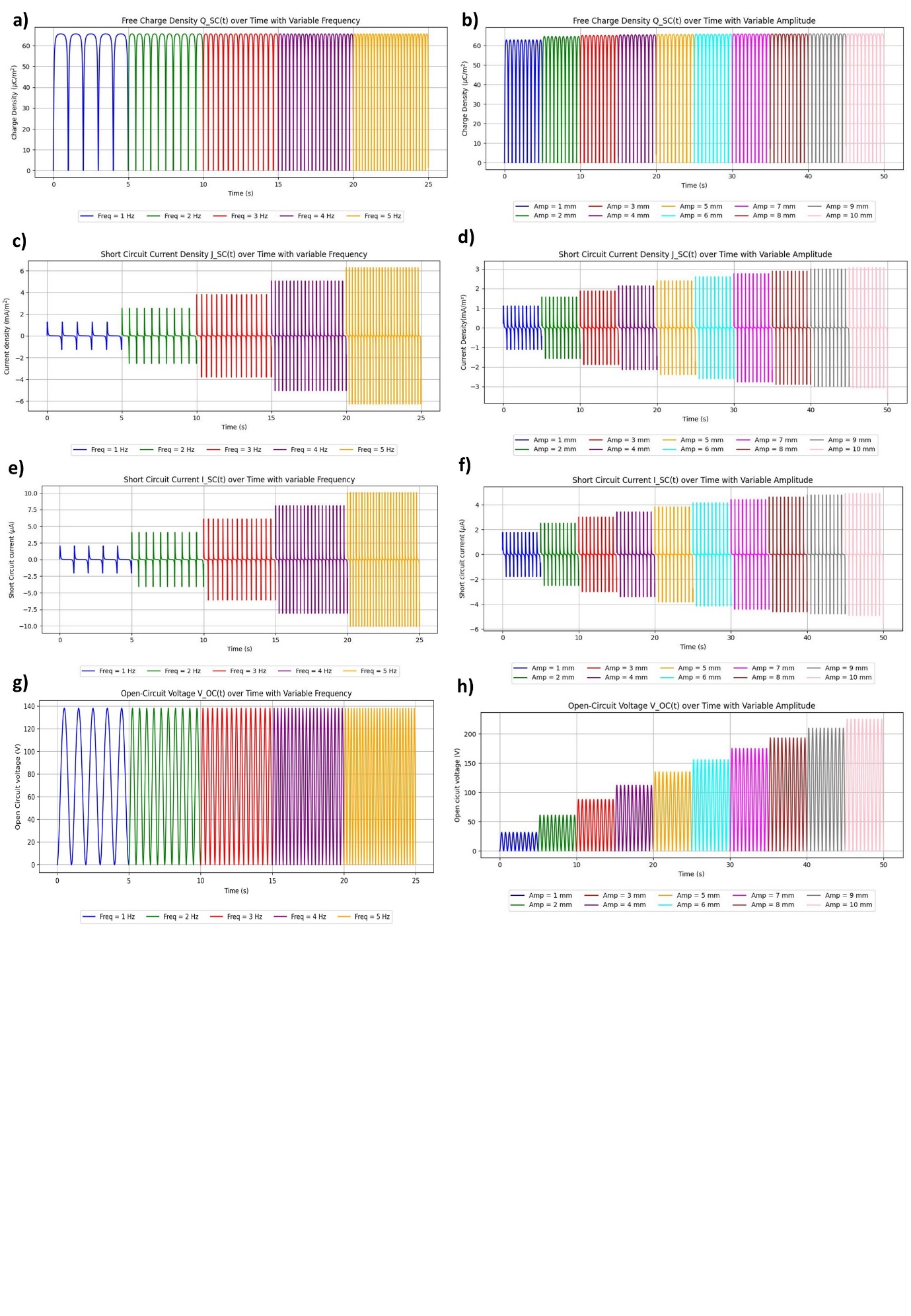


Figure S5: DDEF model simulated results. Charge density changes with a) frequency and b) amplitude, Current density changes with c) frequency and d) amplitude, Short circuit current changes with e) frequency and f) amplitude, Open circuit voltage changes with g) frequency and h) amplitude.

**Supplementary Note 5**

**Contact and Separation Mechanism for TENG Characterization**

A linear actuator rig was developed to characterize all samples. Two copper electrodes were positioned on the insulators to function as the active electrodes. The insulators, with dimensions of 60 × 60 mm, were centrally aligned, used to ensure optimal sample alignment and contact. Standard probes supplied with the equipment were utilized to collect electrical signals from the samples during testing.


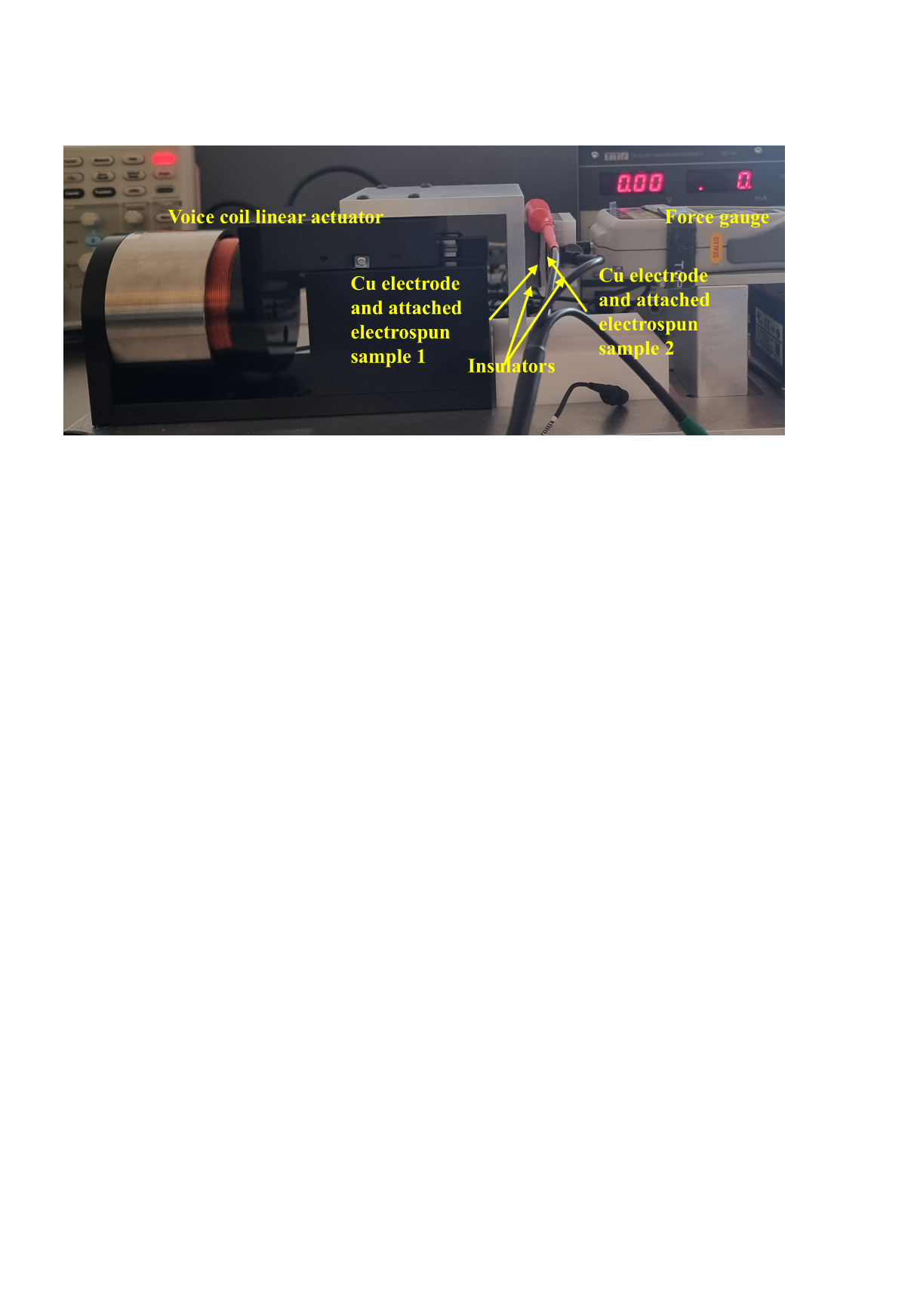


Figure S6: Bespoke set-up used for contact and separation motion.

**Supplementary Note 6**

**RMS voltage base power characterization.**


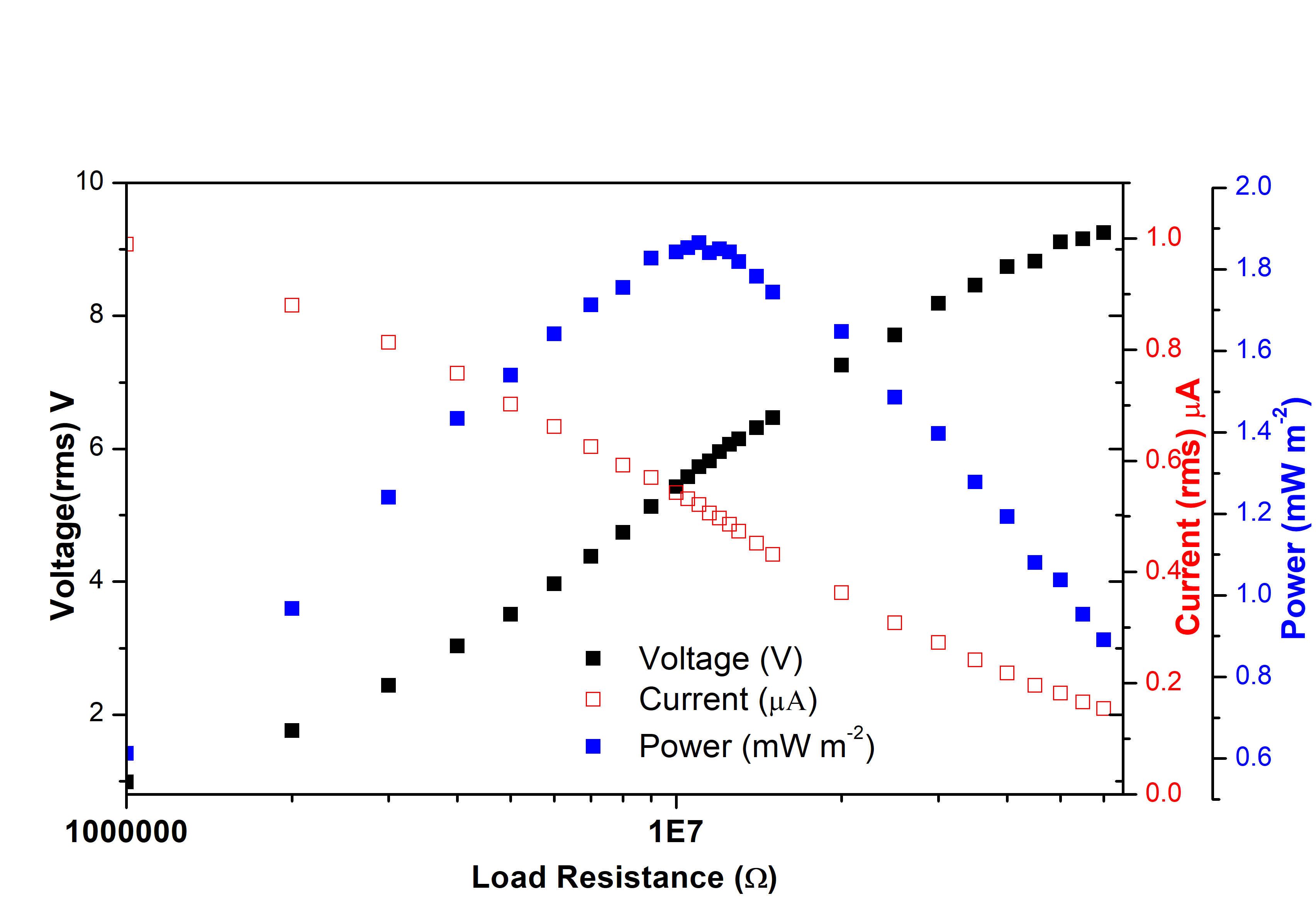


Figure S7: Power generation capability of the optimised TENG as a function of external impedance. (Measured using voltage rms to find peak average power)

**Supplementary Note 7**

**Power conversion efficiency**

Method was followed as described in ref [10]

**Mechanical input power**

Assuming x m elastic compression when tapping.

Taking 10 N as the applied peak force (F_peak_) at the compression of x m the average force during the compression can be calculated

$Averge force (F_{avg})=\frac{F_{peak}}{2}$

For tapping frequency (f) of 2 Hz. The mechanical power

Input work done - $\int Fds= f\times F_{avg}\times x=2\times\frac{10}{2}\times x=10x$

**Electrical output power**

The instantaneous power density is 0.029 W m^-2^ resulting over an active area of 16 cm^2^.

Instantaneous electrical output work = 0.029W $\times$ 16$\times$10^-4^ = 4.64 $\times$ 10^-5^

Power conversion efficiency = $\frac{Electrical output}{Mechanical input} \times100\%$ = $\frac{4.64\times{10}^{-5}}{10x} \times100\%$

Figure S14 shows the parametric sweep of compression vs power conversion efficiency. The lower limit of compression was selected as 5 μm to have a practical power conversion efficiency of bellow 100%.

Some examples : x= 5 μm ; efficiency $\approx$ 92.8%

x= 10 μm ; efficiency $\approx$ 46.4%

x= 50 μm ; efficiency $\approx$ 9.28%

x= 100 μm ; efficiency $\approx$ 4.64%


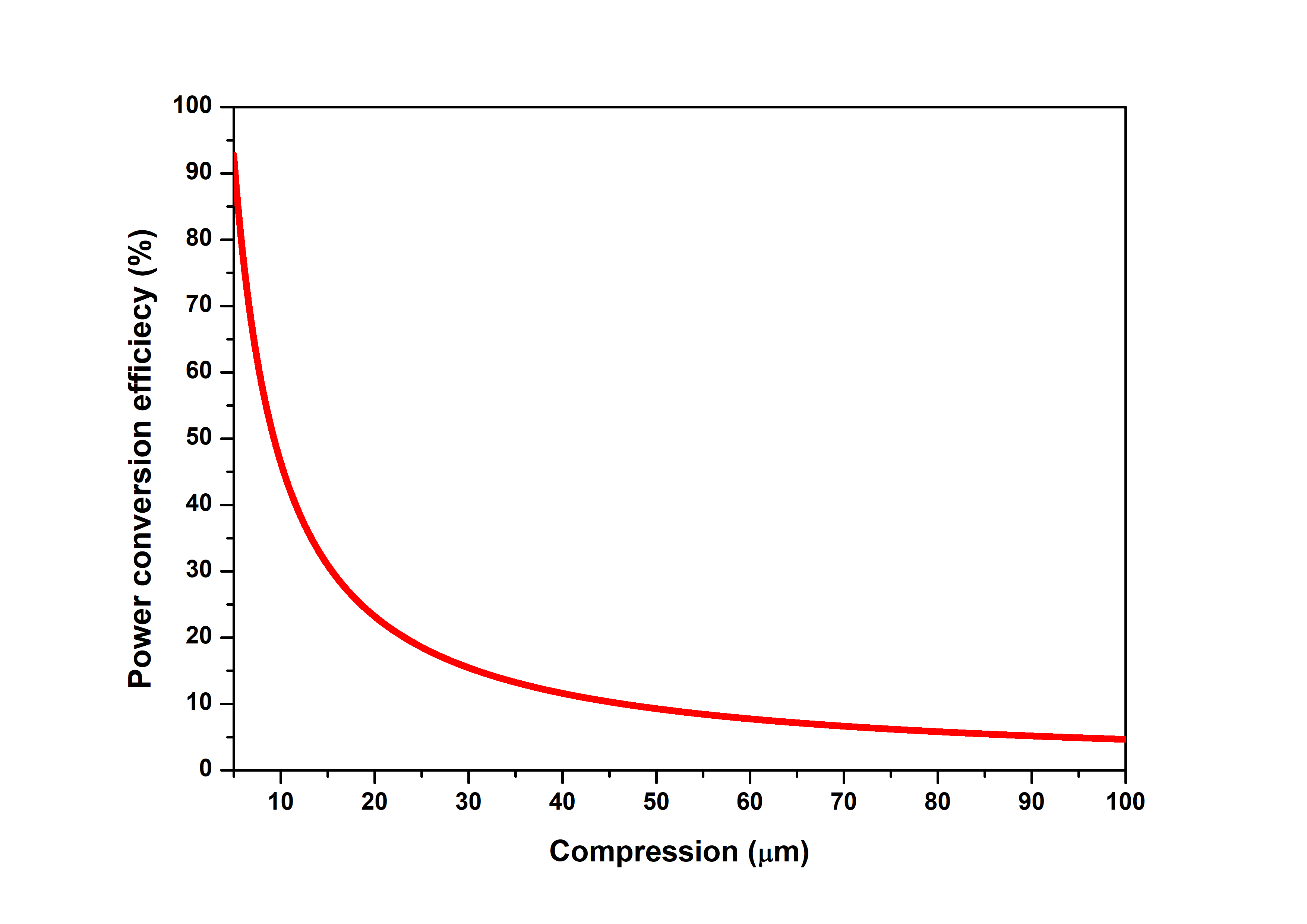


Figure S8: Power conversion efficiency as a function of the elastic compression of the triboelectric layers.

**Supplementary Note 8**

**Stability analysis of develop TENG**


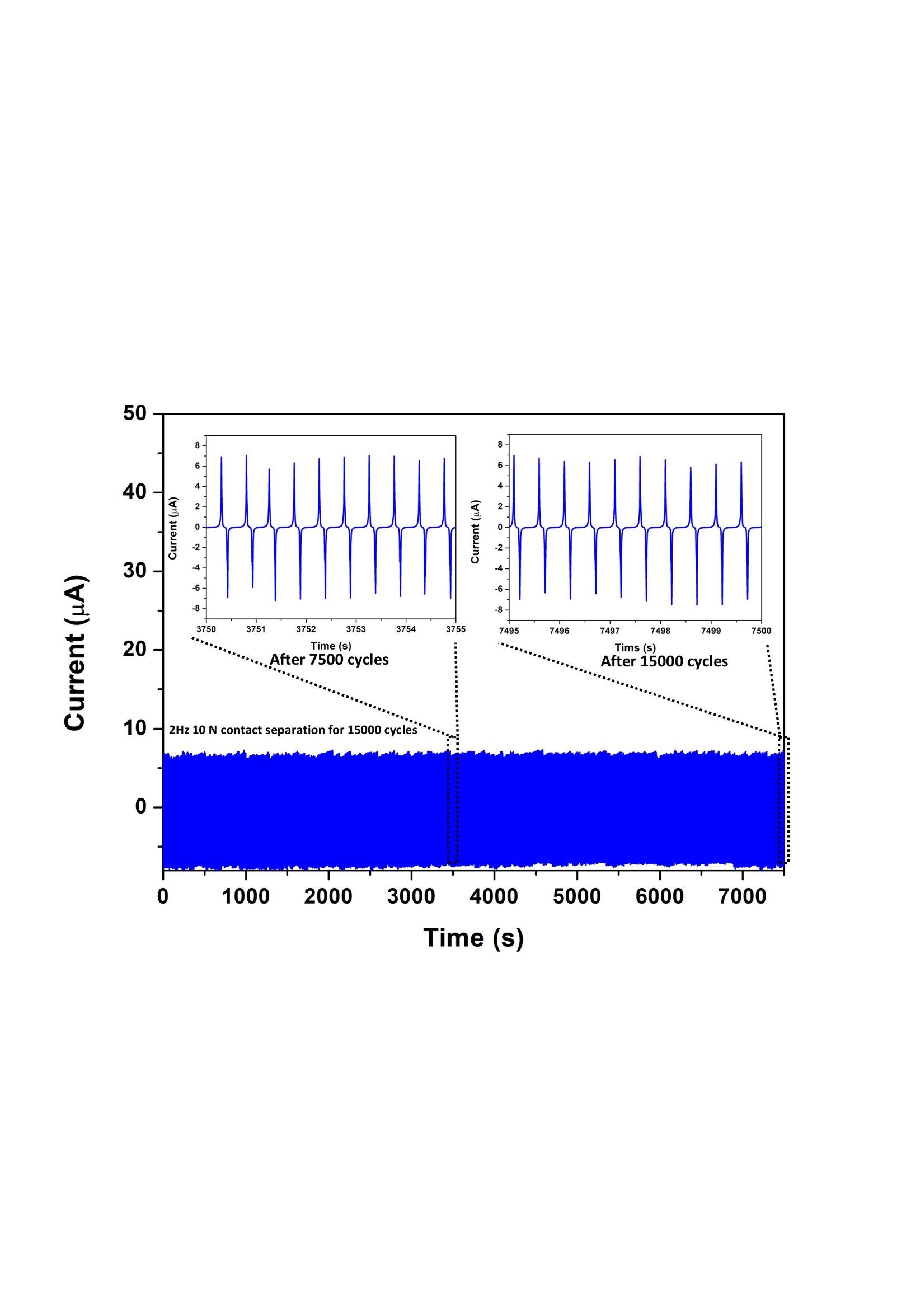


Figure S9: Stability analysis for 15000 contact and separation motion with 2Hz 10 N force.

**Supplementary Note 9**

**
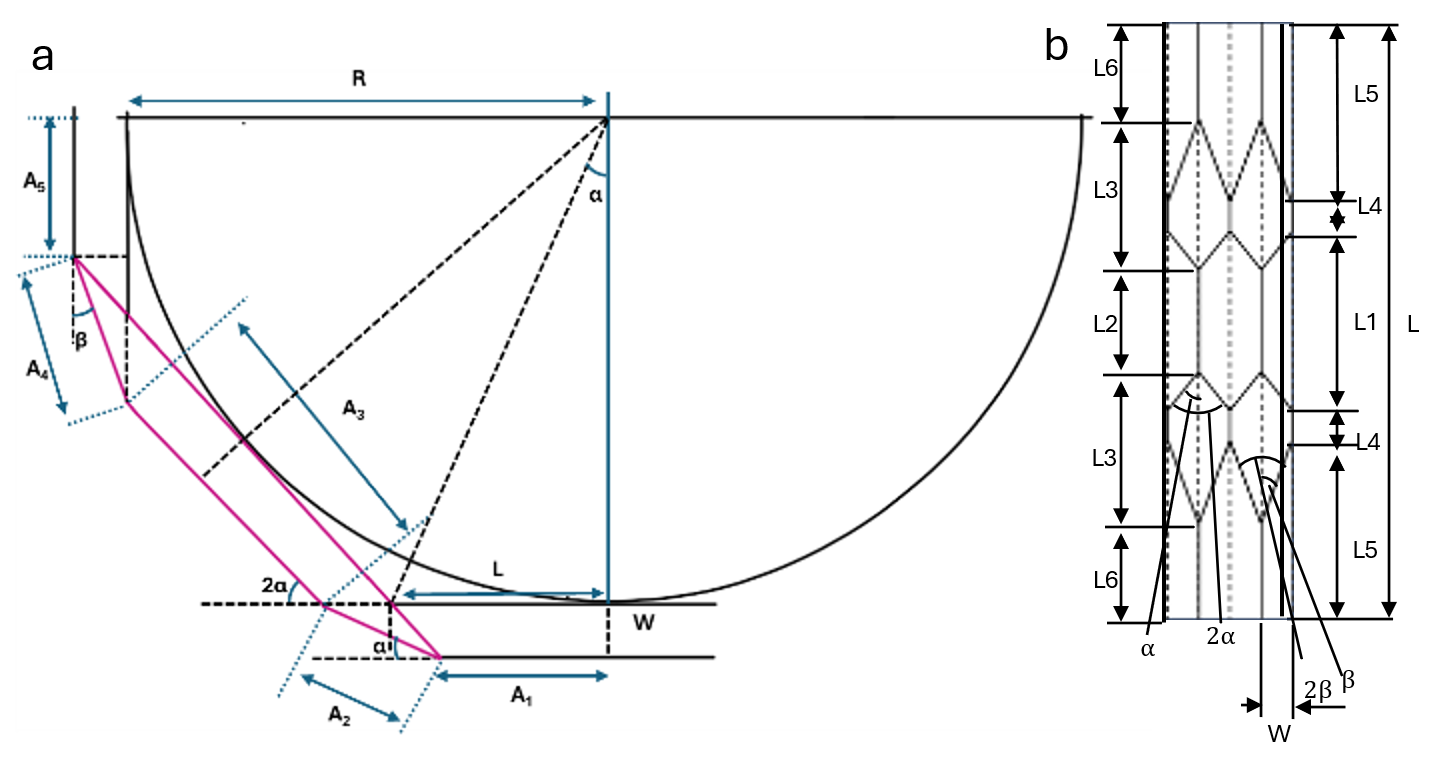
Determining the angles for fabrigami sensor development.**

Figure S10: Model used to develop angles for fabrigami sensor. a) Knee model (taking semi circular architecture. and related bending parts of the fabrigami sensor. b) relevant length, width and angle notations with respect to the fabrigami sensor on the paper.

R- Radius of the knee

W- Width of the fabric ridge panel

A_1_- Half distance of the centre panel

A_2_ – Distance of the first bending

A_3_ – Distance of the side straight part

A_4_ – Distance of the side bending

A_5_ – Vertical panel distance

W – total width of the fabrigami sensor sensing area.

By geometry we can prove

$\beta= \frac{\pi}{4}-\alpha$ (SE10)

**In this we are considering R and W are constants for a given design.**

Based on that

$\boldsymbol{A}_{\boldsymbol{2}}\boldsymbol{=}\frac{\boldsymbol{W}}{\sin\boldsymbol{\propto}}$ **(SE11)**

$A_{4}=\frac{W}{\sin\beta}$ (SE12)

$\boldsymbol{A}_{\boldsymbol{4}}\boldsymbol{=}\frac{\boldsymbol{W}}{\sin\left( \frac{\boldsymbol{\pi}}{\boldsymbol{4}}\boldsymbol{-\alpha} \right)}$ **(SE13)**

$A_{1}=L-\frac{W}{\tan2\alpha}$ (SE14)

$L=R\tan\alpha$ (SE15)

$\boldsymbol{A}_{\boldsymbol{1}}\boldsymbol{=R}\tan\boldsymbol{\alpha}\boldsymbol{-W}\cot\boldsymbol{2}\boldsymbol{\alpha}$ **(SE16)**

$R=A_{1}+A_{2}\cos\alpha+A_{3}\cos2\alpha=R\tan\alpha-W\cot2\alpha+W\cot\alpha+A_{3}\cos2\alpha$ (SE17)

$\boldsymbol{A}_{\boldsymbol{3}}\boldsymbol{=}\frac{\boldsymbol{R-(R}\tan\boldsymbol{\alpha}\boldsymbol{-W}\cot\boldsymbol{2}\boldsymbol{\alpha} \boldsymbol{+W}\cot\boldsymbol{\alpha}\boldsymbol{)}}{\cos\boldsymbol{2}\boldsymbol{\alpha}}$ **(SE18)**

$A_{5}=R-A_{4}\cos\beta-A_{3}\sin2\alpha$ (SE19)

$\boldsymbol{A}_{\boldsymbol{5}}\boldsymbol{=R-W}\cot\left( \frac{\boldsymbol{\pi}}{\boldsymbol{4}}\boldsymbol{-\alpha} \right)\boldsymbol{-}\boldsymbol{(R-}\left( \boldsymbol{R}\tan\boldsymbol{\alpha}\boldsymbol{-W}\cot\boldsymbol{2}\boldsymbol{\alpha} \boldsymbol{+W}\cot\boldsymbol{\alpha} \right)\boldsymbol{)}\tan\boldsymbol{2}\boldsymbol{\alpha}$ **(SE20)**

Based on the Figure s16 b

$L1=2A_{1}+2A_{2}\cos\alpha$ (SE21)

$\boldsymbol{L}\boldsymbol{1=2}\left( \boldsymbol{R}\tan\boldsymbol{\alpha}\boldsymbol{-W}\cot\boldsymbol{2}\boldsymbol{\alpha} \right)\boldsymbol{+2}\boldsymbol{W}\cot\boldsymbol{\alpha}$ **(SE22)**

$L2=2A_{1}$ (SE23)

$\boldsymbol{L}\boldsymbol{2=2(R}\tan\boldsymbol{\alpha}\boldsymbol{-W}\cot\boldsymbol{2}\boldsymbol{\alpha}\boldsymbol{)}$ **(SE24)**

$L3=A_{4}\cos\beta+A_{3}+A_{2}\cos\alpha$ (SE25)

$\boldsymbol{L}\boldsymbol{3=W}\cot\left( \frac{\boldsymbol{\pi}}{\boldsymbol{4}}\boldsymbol{-\alpha} \right)\boldsymbol{+}\frac{\boldsymbol{R-(R}\tan\boldsymbol{\alpha}\boldsymbol{-W}\cot\boldsymbol{2}\boldsymbol{\alpha} \boldsymbol{+W}\cot\boldsymbol{\alpha}\boldsymbol{)}}{\cos\boldsymbol{2}\boldsymbol{\alpha}}\boldsymbol{+W}\cot\boldsymbol{\alpha}$ **(SE26)**

$L4=A_{3}$ (SE27)

$\boldsymbol{L}\boldsymbol{4=}\frac{\boldsymbol{R-(R}\tan\boldsymbol{\alpha}\boldsymbol{-W}\cot\boldsymbol{2}\boldsymbol{\alpha} \boldsymbol{+W}\cot\boldsymbol{\alpha}\boldsymbol{)}}{\cos\boldsymbol{2}\boldsymbol{\alpha}}$ **(SE28)**

$\boldsymbol{L}\boldsymbol{5=R-}\boldsymbol{(R-}\left( \boldsymbol{R}\tan\boldsymbol{\alpha}\boldsymbol{-W}\cot\boldsymbol{2}\boldsymbol{\alpha} \boldsymbol{+W}\cot\boldsymbol{\alpha} \right)\boldsymbol{)}\tan\boldsymbol{2}\boldsymbol{\alpha}$ **(SE29)**

$L6=A_{5}$ (SE30)

$\boldsymbol{L}\boldsymbol{6=R-W}\cot\left( \frac{\boldsymbol{\pi}}{\boldsymbol{4}}\boldsymbol{-\alpha} \right)\boldsymbol{-}\boldsymbol{(R-}\left( \boldsymbol{R}\tan\boldsymbol{\alpha}\boldsymbol{-W}\cot\boldsymbol{2}\boldsymbol{\alpha} \boldsymbol{+W}\cot\boldsymbol{\alpha} \right)\boldsymbol{)}\tan\boldsymbol{2}\boldsymbol{\alpha}$ **(SE31)**

Constraints

$$\alpha<\frac{\pi}{4}$$

Based on the geometry of the joint L, W_1_ and $\alpha$ can be determined. Based on the radius of inner curvature of the knee other distances can be determined.

Taking w = 15 mm knee diameter was taken from ref [26]


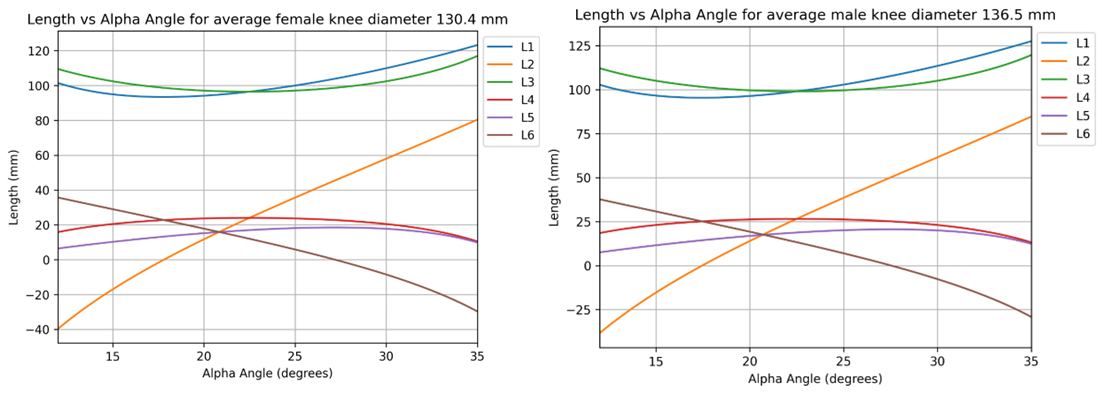


Figure S11: Length vs alpha angle for average female knee diameter of 130.4 mm and average male knee diameter of 136.5 mm.

It should be noted that the knee is not exactly a semi-circle in shape these values need to be corrected after checking the exact knee dimensions.

**Supplementary Note 10**

**Code for the sensor.**

Code can be found at: [**https://doi.org/10.5281/zenodo.16749138**](https://doi.org/10.5281/zenodo.16749138)

**Supplementary Note 11**

**Comparison of fabrigami sensor with relevant range of motion detection based literature.**

| **Sensor type** | **Materials and methods** | **Sensing principle** | **Range of motion** |
| --- | --- | --- | --- |
| Textile resistive sensor (2019)[27] | Silver-coated yarn Agposs T1 sewn into denim fabric which attached to commercial elastic band | Resistance changes with the stretch | Activities such as walking and squatting with 30^0^ motion range |
| Origami-inspired electret-based triboelectric generator (2020)[28] | Two strips made from thick copper/LCP/copper sandwiched composite-with origami structure | Triboelectric self-powered sensing | Elbow bending movement at 90°, 120° and 150°  Arm swinging movement at 30°, 60° and 90°  Hand squeezing  Knee bending movement at 90°, 120° and 150° |
| Embroidered yarn for self-powered sensing and human machine interaction(2022).[29] | Plasma etched copper enamelled with polyurethane yarn and PTFE fabric  Embroidered onto denim fabric | Triboelectric self-powered sensing | Can distinguish walking, running and jumping motions. |
| Low-hysteresis strain sensors using origami-inspired 3D mesostructures (2023)[30] | Origami-inspired foldable 3D mesoscale electrodes, featuring triangular multi-panel thin film on stretchable dielectric substrate with two electrodes | Capacitive field change due to electrode deformation. | Relative capacitances change at 0°, 45°, and 90° with respect to a uniaxial stretching of 70% strain. |
| Kirigami triboelectric spider fibroin microneedle patches for comprehensive joint management (2024)[31] | Spider fibroin microneedles with PU backing and transformed into kirigami cuts. | Triboelectric with microneedles generate signals with bending. | Finger, wrist and elbow bending  Nodding and twisting of head, walking and full flexion of knee |
| Strain Sensor Using Kirigami and Corrugated Structures (2025)[32] | Hydrothermal PZT layers on flexible substrate (embedded in silicone) elastic behaviour achieved with corrugated shape | Piezoelectric strain sensor | Full squat detection, full flexion of knee |
| Fabrigami sensor, 2025 (our work) | Ag-CA electrospun -tribopositive  PVDF electrospun – tribonegative  Attached onto fabrigami substrate | Triboelectric self-powered sensing | Full joint flexion (e.g. 0–90° knee) with 10^0^ movement detection. Distinguishing speed, frequency of different angles, distinguishing different movements, walking, running, squatting and stair climbing. |

**Supplementary note 12**


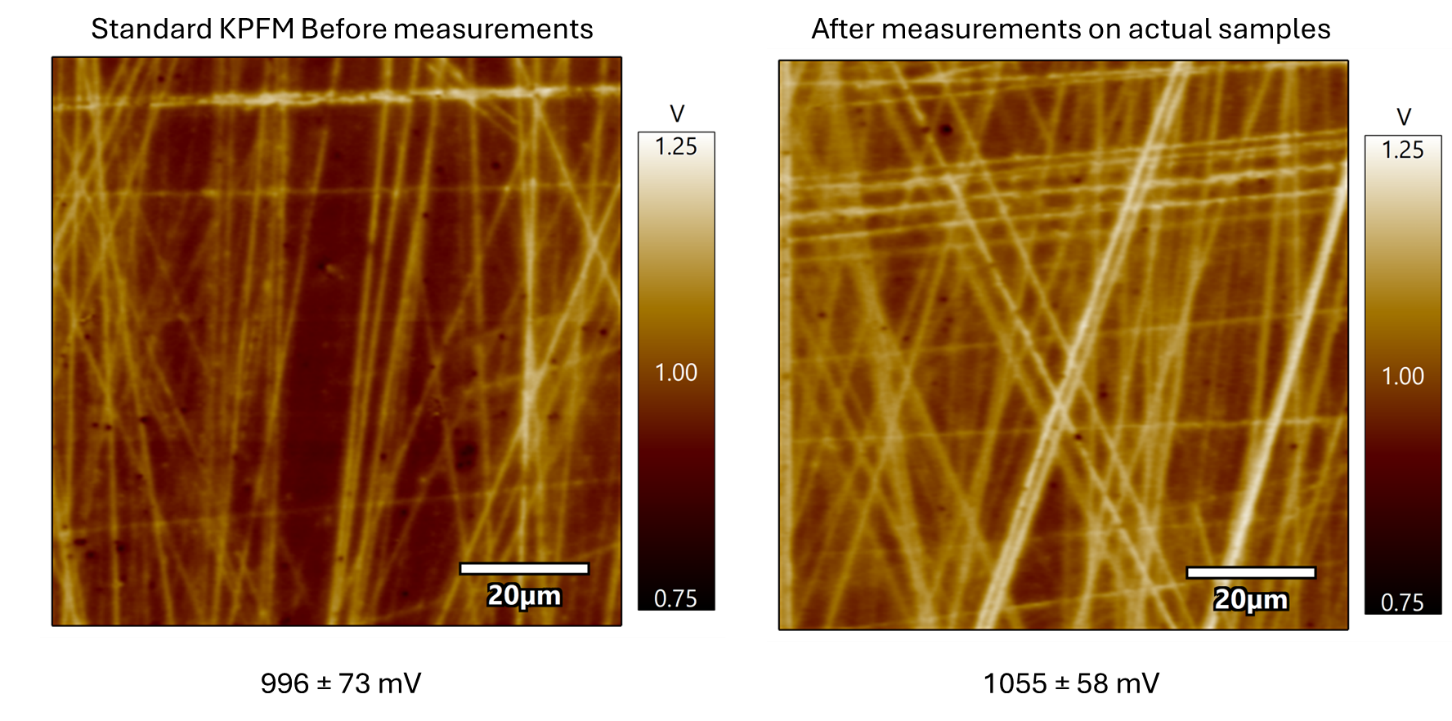


Figure S12: Calibration of the KPFM probe using a standard Au/Al reference sample measured before and after the experiments, confirming probe stability and reliability of the surface potential results.

**Reference**

[1] R. Cao *et al.*, “Self-powered nanofiber-based screen-print triboelectric sensors for respiratory monitoring,” *Nano Res*, vol. 11, no. 7, pp. 3771–3779, Jul. 2018, doi: 10.1007/s12274-017-1951-2.

[2] M.-F. Lin, J. Xiong, J. Wang, K. Parida, and P. S. Lee, “Core-shell nanofiber mats for tactile pressure sensor and nanogenerator applications,” *Nano Energy*, vol. 44, pp. 248–255, Feb. 2018, doi: 10.1016/j.nanoen.2017.12.004.

[3] M. Lou, I. Abdalla, M. Zhu, J. Yu, Z. Li, and B. Ding, “Hierarchically Rough Structured and Self-Powered Pressure Sensor Textile for Motion Sensing and Pulse Monitoring,” *ACS Appl Mater Interfaces*, vol. 12, no. 1, pp. 1597–1605, Jan. 2020, doi: 10.1021/acsami.9b19238.

[4] X. Peng *et al.*, “A breathable, biodegradable, antibacterial, and self-powered electronic skin based on all-nanofiber triboelectric nanogenerators,” *Sci Adv*, vol. 6, no. 26, Jun. 2020, doi: 10.1126/sciadv.aba9624.

[5] Z. Li, M. Zhu, J. Shen, Q. Qiu, J. Yu, and B. Ding, “All-Fiber Structured Electronic Skin with High Elasticity and Breathability,” *Adv Funct Mater*, vol. 30, no. 6, p. 1908411, Feb. 2020, doi: 10.1002/adfm.201908411.

[6] Y. Jiang *et al.*, “UV-Protective, Self-Cleaning, and Antibacterial Nanofiber-Based Triboelectric Nanogenerators for Self-Powered Human Motion Monitoring,” *ACS Appl Mater Interfaces*, vol. 13, no. 9, pp. 11205–11214, 2021, doi: 10.1021/acsami.0c22670.

[7] Y. Shi *et al.*, “Integrated All-Fiber Electronic Skin toward Self-Powered Sensing Sports Systems,” *ACS Appl Mater Interfaces*, vol. 13, no. 42, pp. 50329–50337, Oct. 2021, doi: 10.1021/acsami.1c13420.

[8] S. M. S. Rana *et al.*, “Cation functionalized nylon composite nanofibrous mat as a highly positive friction layer for robust, high output triboelectric nanogenerators and self-powered sensors,” *Nano Energy*, vol. 88, p. 106300, Oct. 2021, doi: 10.1016/j.nanoen.2021.106300.

[9] M. Zhou *et al.*, “Continuously fabricated nano/micro aligned fiber based waterproof and breathable fabric triboelectric nanogenerators for self-powered sensing systems,” *Nano Energy*, vol. 104, p. 107885, Dec. 2022, doi: 10.1016/j.nanoen.2022.107885.

[10] M. T. Rahman, S. S. Rana, M. A. Zahed, S. Lee, E. S. Yoon, and J. Y. Park, “Metal-organic framework-derived nanoporous carbon incorporated nanofibers for high-performance triboelectric nanogenerators and self-powered sensors,” *Nano Energy*, vol. 94, p. 106921, Apr. 2022, doi: 10.1016/j.nanoen.2022.106921.

[11] T. Bhatta *et al.*, “Siloxene/PVDF Composite Nanofibrous Membrane for High-Performance Triboelectric Nanogenerator and Self-Powered Static and Dynamic Pressure Sensing Applications,” *Adv Funct Mater*, vol. 32, no. 25, p. 2202145, Jun. 2022, doi: 10.1002/adfm.202202145.

[12] M. Cui, H. Guo, W. Zhai, C. Liu, C. Shen, and K. Dai, “Template-Assisted Electrospun Ordered Hierarchical Microhump Arrays-Based Multifunctional Triboelectric Nanogenerator for Tactile Sensing and Animal Voice-Emotion Identification,” *Adv Funct Mater*, vol. 33, no. 46, p. 2301589, Nov. 2023, doi: 10.1002/adfm.202301589.

[13] Y. Bai, Z. Zhou, Q. Zhu, S. Lu, Y. Li, and L. Ionov, “Electrospun cellulose acetate nanofibrous composites for multi-responsive shape memory actuators and self-powered pressure sensors,” *Carbohydr Polym*, vol. 313, p. 120868, Aug. 2023, doi: 10.1016/j.carbpol.2023.120868.

[14] J. Yang *et al.*, “High-Performance Flexible Wearable Triboelectric Nanogenerator Sensor by β-Phase Polyvinylidene Fluoride Polarization,” *ACS Appl Electron Mater*, vol. 6, no. 2, pp. 1385–1395, Feb. 2024, doi: 10.1021/acsaelm.3c01678.

[15] M. Robiul Islam *et al.*, “Poly‐DADMAC Functionalized Polyethylene Oxide Composite Nanofibrous Mat as Highly Positive Material for Triboelectric Nanogenerators and Self‐Powered Pressure Sensors,” *Adv Funct Mater*, p. 2403899, May 2024, doi: 10.1002/adfm.202403899.

[16] M. Yasar, P. Hassett, N. Murphy, and A. Ivankovic, “β Phase Optimization of Solvent Cast PVDF as a Function of the Processing Method and Additive Content,” *ACS Omega*, vol. 9, no. 24, pp. 26020–26029, Jun. 2024, doi: 10.1021/acsomega.4c01221.

[17] R. D. I. G. Dharmasena and S. R. P. Silva, “Towards optimized triboelectric nanogenerators,” *Nano Energy*, vol. 62, no. April, pp. 530–549, Aug. 2019, doi: 10.1016/j.nanoen.2019.05.057.

[18] R. D. I. G. Dharmasena, K. D. G. I. Jayawardena, C. A. Mills, R. A. Dorey, and S. R. P. Silva, “A unified theoretical model for Triboelectric Nanogenerators,” *Nano Energy*, vol. 48, no. March, pp. 391–400, Jun. 2018, doi: 10.1016/j.nanoen.2018.03.073.

[19] R. D. I. G. I. G. Dharmasena *et al.*, “Triboelectric nanogenerators: providing a fundamental framework,” *Energy Environ Sci*, vol. 10, no. 8, pp. 1801–1811, 2017, doi: 10.1039/c7ee01139c.

[20] M. M. Rastegardoost, O. A. Tafreshi, Z. Saadatnia, S. Ghaffari-Mosanenzadeh, C. B. Park, and H. E. Naguib, “Porous PVDF mats with significantly enhanced dielectric properties and novel dipole arrangement for high-performance triboelectric nanogenerators,” *Appl Mater Today*, vol. 30, p. 101732, Feb. 2023, doi: 10.1016/j.apmt.2023.101732.

[21] X. Ren *et al.*, “Giant energy storage density in PVDF with internal stress engineered polar nanostructures,” *Nano Energy*, vol. 72, p. 104662, Jun. 2020, doi: 10.1016/J.NANOEN.2020.104662.

[22] “Properties: Cellulose Acetate.” Accessed: Sep. 03, 2025. [Online]. Available: https://www.azom.com/properties.aspx?ArticleID=1461

[23] F. Zhang, X. Li, Z. yuan Lan, N. Zhang, J. hui Yang, and Y. Wang, “Sandwich-structured cellulose acetate dielectric films toward high-temperature energy storage application,” *Carbohydr Polym*, vol. 366, p. 123934, Oct. 2025, doi: 10.1016/J.CARBPOL.2025.123934.

[24] Z. M. Dang, J. K. Yuan, J. W. Zha, T. Zhou, S. T. Li, and G. H. Hu, “Fundamentals, processes and applications of high-permittivity polymer–matrix composites,” *Prog Mater Sci*, vol. 57, no. 4, pp. 660–723, May 2012, doi: 10.1016/J.PMATSCI.2011.08.001.

[25] N. Ahmadi, G. Rezazadeh, A. Rahmani, and M. Ghanbari, “Analyzing the effects of polymeric dielectric materials on micro capacitive pressure sensors: A model incorporating displacement-dependent porosity,” *Heliyon*, vol. 10, no. 9, p. e30626, May 2024, doi: 10.1016/J.HELIYON.2024.E30626/ASSET/D066C398-1E36-4B4F-952E-768B8AF5B29D/MAIN.ASSETS/GR19.JPG.

[26] D. Sieroń *et al.*, “Knee Diameter and Cross-Section Area Measurements in MRI as New Promising Methods of Chondromalacia Diagnosis-Pilot Study,” *Medicina (Lithuania)*, vol. 58, no. 9, p. 1142, Aug. 2022, doi: 10.3390/medicina58091142.

[27] S. Hu, M. Dai, T. Dong, and T. Liu, “A textile sensor for long durations of human motion capture,” *Sensors (Switzerland)*, vol. 19, no. 10, p. 2369, May 2019, doi: 10.3390/s19102369.

[28] K. Tao *et al.*, “Origami-inspired electret-based triboelectric generator for biomechanical and ocean wave energy harvesting,” *Nano Energy*, vol. 67, p. 104197, Jan. 2020, doi: 10.1016/j.nanoen.2019.104197.

[29] Y. Chen *et al.*, “Flexible, durable, and washable triboelectric yarn and embroidery for self-powered sensing and human-machine interaction,” *Nano Energy*, vol. 104, p. 107929, Dec. 2022, doi: 10.1016/j.nanoen.2022.107929.

[30] X. Huang *et al.*, “High-stretchability and low-hysteresis strain sensors using origami-inspired 3D mesostructures,” *Science Advances*, vol. 9, no. 34, Aug. 2023, doi: 10.1126/sciadv.adh9799.

[31] S. Li, S. Cao, H. Lu, B. He, and B. Gao, “Kirigami triboelectric spider fibroin microneedle patches for comprehensive joint management,” *Materials Today Bio*, vol. 26, p. 101044, Jun. 2024, doi: 10.1016/j.mtbio.2024.101044.

[32] C. Das and G. H. Feng, “A High-Performance Directional Strain Sensor Using Kirigami and Corrugated Structures for Real-Time Knee Joint Movement Detection,” *IEEE Sensors Journal*, vol. 25, no. 7, pp. 10775–10783, 2025, doi: 10.1109/JSEN.2025.3541672.
